# Morphological Assessment and Biomarkers of Low-Grade, Chronic Intestinal Inflammation in Production Animals

**DOI:** 10.3390/ani12213036

**Published:** 2022-11-04

**Authors:** Igor Soares, Bruna L. Belote, Elizabeth Santin, Gabriela C. Dal Pont, Michael H. Kogut

**Affiliations:** 1 ISI Institute, Cambé, Parana 86187-025, Brazil; 2 Department of Poultry Science, Texas A&M University, College Station, TX 77843, USA; 3 Southern Plains Agricultural Research Center, United States Department of Agriculture—Agricultural Research Service (USDA-ARS), College Station, TX 77845, USA

**Keywords:** low-grade inflammation, intestinal biomarkers, immunometabolism, gut integrity

## Abstract

**Simple Summary:**

Production animals are continuously exposed to environmental and dietary factors that might induce a state of low-grade, chronic intestinal inflammation. This condition compromises the productive performance and well-fare of these animals, requiring studies to understand what causes it and to develop control strategies. An intestinal inflammatory process is generally associated with alterations in the structure and functionality of its wall, resulting in the release of cellular components into the blood and/or feces. These components can act as biomarkers, i.e., they are measured to identify and quantify an inflammatory process without requiring invasive methods. In this review we discuss the mechanisms of low-grade inflammation, its effects on animal production and sustainability, and the identification of biomarkers that could provide early diagnosis of this process and support studies of useful interventional strategies.

**Abstract:**

The complex interaction between the intestinal mucosa, the gut microbiota, and the diet balances the host physiological homeostasis and is fundamental for the maximal genetic potential of production animals. However, factors such as chemical and physical characteristics of the diet and/or environmental stressors can continuously affect this balance, potentially inducing a state of chronic low-grade inflammation in the gut, where inflammatory parameters are present and demanding energy, but not in enough intensity to provoke clinical manifestations. It’s vital to expand the understanding of inflammation dynamics and of how they compromise the function activity and microscopic morphology of the intestinal mucosa. These morphometric alterations are associated with the release of structural and functional cellular components into the feces and the blood stream creating measurable biomarkers to track this condition. Moreover, the identification of novel, immunometabolic biomarkers can provide dynamic and predictors of low-grade chronic inflammation, but also provide indicators of successful nutritional or feed additive intervention strategies. The objective of this paper is to review the mechanisms of low-grade inflammation, its effects on animal production and sustainability, and the biomarkers that could provide early diagnosis of this process and support studies of useful interventional strategies.

## 1. Introduction

The gastrointestinal tract (GIT) is of great importance in animal production, not simply because of it is role in digestion and absorption of nutrients, but also due to its immunometabolic and neuroendocrinologic functions in regulating animal physiology [1,2,3,4]. The intestine of all animals is a complex, but hollow tube in which the external environment within the lumen is separated from the inside of the animal by a thin, yet remarkably efficient barrier system. The lumen side of the intestine harbors a diverse community of commensal microbes that have co-evolved with the host immune system which is predominately located in the internal side of the intestine. These microbes, tolerated and anatomically contained by the mucosal immune system, execute functions that are critical for host physiology.

The immune system contributes to the intestinal homeostasis by diverse ways. Besides defending the organism against potential pathogens, the immune system prevents organisms of the microbiota from reaching the intestinal epithelium or deeper tissues and develops a state of tolerance to the commensal microorganisms of the lumen [5]. This constant vigilance of the microbiota involves physical, biochemical, and immunological barriers of the intestinal mucosa [5]. During intestinal homeostasis, the microbiota and the immune system operates in symbiosis through a bi-directional relationship, where both characters influence one another’s development, composition, and functionality [6]. The tolerance required to maintain this state of homeostasis involves the activation of pattern recognition receptors (PRRs) present in certain cells of the innate immune system. These receptors are activated by their continuous interaction with structural and functional elements from the gut microbial community, including lipopolysaccharides (LPS) and peptidoglycans (PGNs) from the bacterial cell walls and other microbial-associated molecular patterns (MAMPs). Cells containing these receptors present PRR-mediated signaling pathways that, when activated, regulates the responsiveness of PRRs, induce the production of high levels of regulatory proteins, such as interleukin-10 (IL-10) and transforming growth factor-β (TGF-β), and induce the differentiation of the T-regulatory subpopulation of T lymphocytes [5,7].

The ‘intestinal ménage trois’, composed by the gut microbiota, mucosal immune system, and diet, represent a multidirectional, chemical, and biological interactive complex. These elements work together, influencing each other at the cellular and molecular level to coordinate an intestinal immunometabolic homeostasis [8] that is translated as a ‘healthy’ gut. The nutritional components and physical form of an animals’ diet, as well as its presence or not (e.g., starving, increased feed intakes and nutrient excesses) on the intestinal lumen have profound effects not only on gut health, but also on the total energy homeostasis and overall health of the animal [9,10]. In the context of this review, the microbiota transduces dietary nutrients into signals recognized by the PRRs of the innate immune system to promote or inhibit inflammation [10]. This process is mediated by the metabolic reprogramming of immune cells, merging immunological and metabolic processes into a process called immunometabolism and that is intimately associated with the gut and organismal health [11,12,13,14,15].

Historically, metabolism and inflammation have been viewed as two separate physiological processes with distinct but essential roles in animal biology: metabolism regulates nutrient uptake and usage while inflammation is a protective immune response involved in host defense and repair. Both systems react to a host’s environmental stressors to restore homeostasis. The interplay between metabolic status and immune response (immunometabolism) plays an important role in maintaining homeostasis, understanding disease pathogenesis, and developing novel disease interventions in the context of the metabolic regulation of immune functionality.

Recognizing the immunometabolic pathways and their morphological, cellular, and biochemical components will help to identify, characterize, and standardize specific biomarkers that are accurate indicators of normal or pathogenic processes in production animals.

## 2. Intestinal Inflammation: What Is It?

The intestinal inflammation participates in dysbiosis in poultry and swine [16,17], but more studies are necessaire for us to comprehend if the inflammation is the cause or the effect of this condition [18,19,20,21]. However, both acute and chronic inflammation are known to cause dysbiosis, irrespective if the process derives from infection or not (“sterile inflammation”) [17]. The disruption of GIT homeostasis can jeopardize animals’ health thus affecting performance and profitability. Along with the complexity of the organ, several external factors affect the intestinal homeostatic status and stimulates inflammation. These factors include thermal and behavioral stress, nutritional challenges, animal density and pathogen exposure [18,22,23].

Inflammation is a normal and highly regulated physiological reaction that aims to recover and repair a harmed tissue. In response to the detection of infectious processes and damage to host structures, cells of the innate immunity produce signaling molecules that activate a network of immunological and physiological events for the purpose of restoring the homeostasis and functionality [24,25,26]. Varied factors can trigger an inflammatory response, shaping its phenotype, efficacy, duration, and consequences. The physiological inflammation, one of the possible phenotypes, is the balance between tolerance to the microbiota and reactivity to pathogens, as we discuss in our introduction to this review. The pathologic inflammation is usually an acute process that occurs in response to toxins and infection, commonly leading to collateral damage to the inflamed site and its surrounding, as well as to increased metabolic energy use. The metabolic inflammation is a chronic low-grade response induced by the excessive nutrient intake, where the consequent metabolic surplus activates the same signaling pathways that operate in immune responses to pathogens. (vi) Sterile inflammation, also a chronic low-grade inflammatory process, occurs in the absence of infection as a response to chemical, physical, and metabolic stimuli [24].

The physiological inflammation is an essential component of the intestinal homeostasis, but other inflammatory phenotypes must be limited due to their impact on the intestinal functionality and their energetic cost. Animals in the modern production industry are not only threatened by microbiologic challenges, but also by non-infectious factors that can lead to low-graded inflammation, such as increased feed intake/nutrient excess and components in ingredients of the diet that can activate receptors of the immune system. However, the impact of these nutritional triggers can be minimized by thoughtful dietary strategies. These practices include the use of enzymes (e.g., mannanase, proteases, xylanases) to reduce undigestible dietary components (e.g., mannans and other non-starch polysaccharides from the cell wall of vegetable ingredients) that may be recognized by PRRs or be fermented by the microbial community, resulting in the production of pro-inflammatory metabolites.

## 3. Inflammation Impact on Animal Production

Although not constituting a clinical disease, an excessive or chronically activated inflammatory response contributes to loss of tissue function and waste of metabolic energy, leading to poor performance and decreased animal welfare [25]. Gut inflammation promotes drastic alterations on intestinal architecture, resulting in increased intestinal permeability [27,28,29], decreased absorption and digestibility of nutrient, occurrence of fluid loss and diarrhea [23,28,29]. The increase in the permeability of the intestinal mucosa, also referred as “leaky gut”, leads to translocation of gut bacteria, microbial compounds, and/or antigens to the blood stream, generating a systemic immune response [23,27,28,29,30].

Besides diverting nutrients and energy from functions that are essential to the animal productivity, the inflammatory process can also induce tissue catabolism [31,32,33]. As an example, proteins in the skeletal muscles undergo proteolysis and protein synthesis is limited to increase the availability of amino acids to fuel the inflammatory responses [34]. Lastly, the metabolism of lipids and sugars stores are increased to produce more energy for the immune system [31].

## 4. Chronic Inflammation

In his recent essay, Medzhitov [35] stated that “because infection and injury-induced inflammation are the most prominent and most studied forms of the response, we may have skewed our understanding of inflammation according to these extreme conditions”. In commercial animal production, this reductionist view of inflammation and the use of antibiotic growth promoters (AGPs) in animal feeds limited our understanding of the complete physiological roles of inflammation in maintaining and monitoring tissue homeostasis [24,25]. Specifically, the removal of AGPs from animal feeds has provided evidence that the failure to remove the inflammatory trigger results in a chronic and low-grade sterile inflammation. As mentioned above, this basal inflammatory response impairs the ability of the animal to reach 100% of its genetic potential given that nutrients are diverted away from growth to support the inflammatory response.

Currently, commercial swine and broiler operations are regularly exposed to several non-infectious, environmental factors that can act as inflammatory triggers, including animal density, poor quality of feed ingredients, nutrient dense diets (high protein or high non-starch polysaccharides), changes in feed formulation, mycotoxins, diets with high amounts of antinutritional factors, and heat stress. The exposure to any of these triggers for an extended period (days to weeks) can lead to the above-mentioned chronic intestinal inflammatory state, resulting in a disrupted digestive function, increased oxidative stress, microbiota dysbiosis, loss of barrier functionality, and immune dysfunctions, as we described in our study on the development of models to induce low-graded intestinal inflammation [36]. Additionally, some swine and broiler producers have relied on antibiotic growth promoters AGPs to compensate for potential poor husbandry and management issues. Therefore, AGPs were never truly used to control enteric infections, as stated by Dal Pont et al. [36] given that intestinal infections predominately promote acute but not chronic inflammation.

## 5. Morphometric Assessment of Gut Health

Morphometric analysis is one of the possible methods to evaluate gut health in animal production and it becomes even more important during the occurrence of low-grade chronic inflammation, where lesions in the intestine are not visible to the naked eye. Dal Pont and colleagues clearly demonstrated this in their development of a diet-triggered low-grade, chronic inflammation model in broiler chickens, where no macroscopic lesions were observed throughout the 35-days growth cycle [36]. A robust characterization of the microscopic processes constituting this continuous basal inflammation becomes essential, as long as we understand that its negative impacts on animal performance starts long before macroscopic alterations are observed, if at all.

For many years, the microscopic analysis of gut health has been based on the measurement of intestinal villus height and crypt depth [37]. Briefly, the villi function as absorptive structures and their height has been indicative of the absorptive area for nutrients. Whereas, the crypts are the primary sites of epithelial progenitor cells, with their depth expressing the turnover rate of the intestinal epithelium [38]. Thus, a higher villus enhances the absorptive capacity of the lumen and deeper crypts indicate a rapid epithelial cell turnover because various types of special cells are present in the crypt, including absorptive, secretory, and regenerative cells [38]. Although these two morphological features may provide an indication of structural alterations in the intestines, they provide no real direct functional evaluations of them. In fact, the recognition of MAMPS and damage-associated molecular patterns (DAMPS) by PRRs releases a cascade of chemical mediators that will alter the morphology of the intestinal mucosa. Consequently, several microscopic alterations may be observed during this process, including increases in lamina propria thickness, epithelial thickness, enterocytes proliferation, inflammatory cell infiltration in the epithelium, inflammatory cell infiltration in the lamina propria, goblet cells proliferation, and congestion of villus by blood cells and could increase the surface area of the villi that is inverse to its functionality.

In broilers, the histological alterations that succeed the macroscopic lesions lead to the development of the I See Inside (ISI) methodology [39,40,41,42,43]. This tool has gained attention by translating microscopic changes in numbers that can be correlated to zootechnical performance and by characterizing the health and functionality of the intestinal mucosa based on specific parameters that are compromised under inflammation. Moreover, the ISI methodology has supported the characterization of sub-clinical inflammatory processes that might not cause gross lesions in all upper intestine and cecum. In studies applying the methodology, broilers fed with low levels of aflatoxin (50 to 250 ppb) in experimental conditions did not present any macroscopic lesions in targeted organs, although histopathological changes at the same ages were observed [39,44,45]. Applying a model of enteritis elicited by *Clostridium perfringens* and *Eimeria* spp., Sanches et al. [43] did not observe gross lesions in the challenged broilers, but microscopic alterations were indeed verified in the intestinal mucosa these animals. Interestingly, the unchallenged birds presented a similar (but statistically lower) pattern in the evolution of the inflammatory parameters when compared to the infected group. This finding suggested that the experimental challenge just worsened an already ongoing low-grade inflammation in the gut mucosa, which could be linked to dietary, management, and/or environmental factors, as cited above. In fact, the ISI methodology allowed the researchers to differentially quantify the histological inflammatory lesions between groups and to statistically correlate them with zootechnical performance.

**Figure 1:**
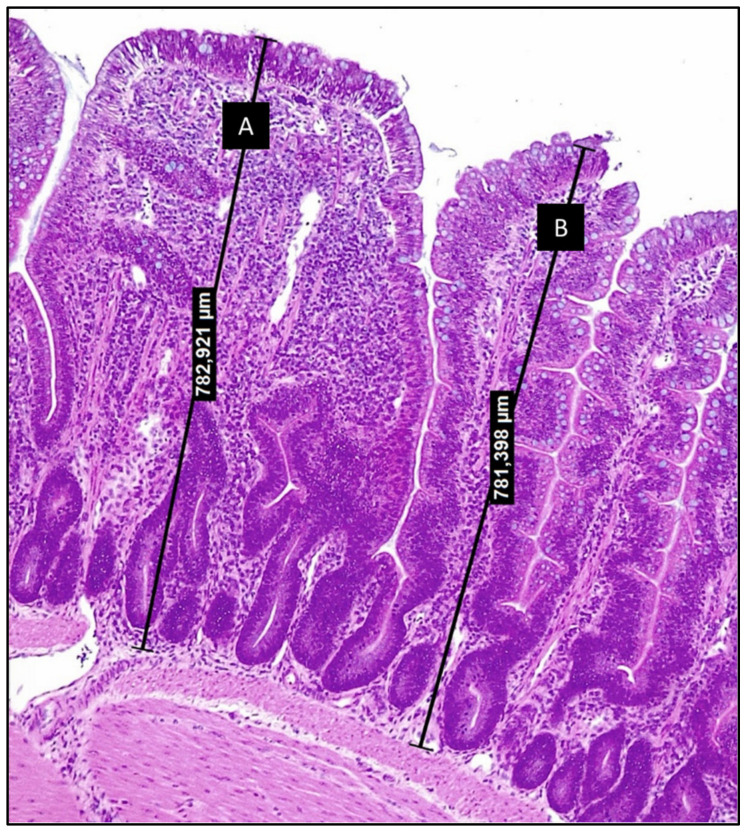
Photomicrography of chicken ileum section stained with hematoxylin and eosin (200×) (from authors archive). Villi A and B display very close heights, although villus A presents increased lamina propria thickness and increased infiltration by inflammatory cells. These alterations are comprised by the ISI methodology and allow to relate structural alterations to the loss of functionality.

Correlations between the villus width and the ISI scores (data not published) suggest that the inflammatory process (expressed through the ISI methodology) turns the villus larger, although the expanded surface of these structures does not mean a better absorptive capacity. In fact, villi can present very close heights but with different levels of internal alterations related to inflammation (Figure 1). In broilers, the parameters measured by the ISI methodology have better characterized the losses of functionality in the whole intestine. The chronic inflammation characterized by the ISI scores at initial ages was statistically correlated with lower zootechnical performance at later periods [41].

Two novel models to induce low-grade gut inflammation were applied by Dal Pont et al. [36] in broilers chickens with the ISI methodology as a “gold test” of microscopic villi changes. The increased ISI scores of histologic alterations in the duodenal, jejunal, and ileal mucosa were joined by a significant increase in the leak of calprotectin and lipocalin through the feces, important inflammatory biomarkers. In fact, the ISI methodology was able to prove the occurrence of a low-grade inflammation and demonstrated that the chicken intestinal inflammation evolves in a spatial and temporal pattern through the small intestine.

## 6. Structural and Inflammatory Biomarkers

**Figure 2:**
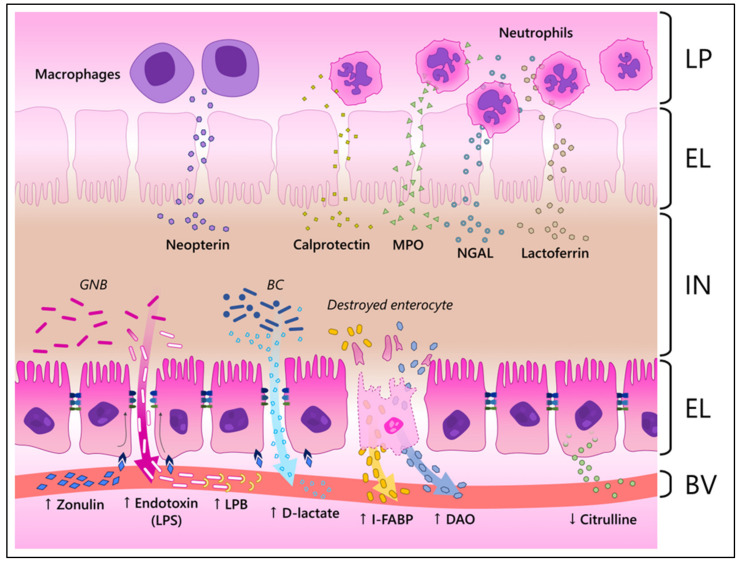
Illustration of how different biomarkers reach the blood and/or the feces during inflammation in the intestinal mucosa. LP, lamina propria; EL, epithelial layer; IN, intestinal lumen; BV, blood vessel; GNB, Gram-negative bacteria; BC, bacterial community; MPO, myeloperoxidase; NGAL, neutrophil-gelatinase associated lipocalin; LPS, lipopolysaccharide; LPB, LPS-binding protein; I-FABP, intestinal fatty acid binding protein; DAO, diamine oxidase. ↑, increased; ↓, decreased levels during inflammation. In the illustration, zonulin interacts with its receptors in enterocytes, changing the structure of the tight junctions and, consequently, increasing the paracellular influx. This increased permeability allows bacterial components and metabolites, such as endotoxins and D-lactate, to cross the epithelial barrier and reach the blood stream. Destroyed enterocytes resultant of the inflammatory process release their internal components, such as I-FABP and DAO, in the circulatory system. Healthy enterocytes produce citrulline and release it into the blood stream. Therefore, its levels are reduced when enterocytes are damaged. Neutrophils and macrophages that reach the mucosa release antimicrobial components such as neopterin, calprotectin, MPO, NGAL and lactoferrin, which reach the IN. It must be noted that the described biomarkers might reach both the IN and BV.

Production animal industries require reliable non-invasive biomarkers of gut health, inflammation, and disease. Two recent reviews have documented a number of potential biomarkers in poultry [3,46]. However, most of the potential biomarkers of animal gut health been inconsistent in documenting a healthy or diseased intestine. Here, we outline a number of potential novel biomarkers that have recently been identified as viable tools to evaluate production animal gut health, but probably will require more samples under different conditions to validate their value to the production animal industry. In addition to the descriptions, Figure 2 illustrates how these components reach the blood and/or feces in a state of inflammation.

We should point out that all the potential biomarkers reviewed herein (particularly the metabolic markers discussed in the next topic) are projected as functional quantitative measurements of intestinal inflammation and are not disease specific. Fecal biomarkers would be more definitive of intestinal inflammation, whereas plasma biomarkers would be indicators of more systemic conditions. Overall, these biomarkers can be predictive and have the potential to serve as a diagnostic screening tests. Each biomarker will require the development of a functional range to determine a ‘threshold’ level that is an indicator of chronic intestinal inflammation. The main applications of these tests would be to rapidly differentiate healthy animals from diseased.

Lipopolysaccharide-binding protein (LPB) is produced by hepatocytes and released to the blood stream as part of the acute phase response to LPS, upregulated by IL-1, IL-6, and tumor necrosis factor α [47]. While a N-terminal domain of LPB binds to the LPS lipid A with high affinity, its C-terminal continues the endotoxin processing by delivering it to (i) CD14 proteins, thus forming a LPS-CD14 complex that is recognized by Toll-like receptors 4 and stimulates the immune response, or (ii) to circulating lipoproteins, which take them inside hepatocytes for metabolizing and excretion on the bile, where it’s deactivated by bile salts [48,49,50,51,52]. Plasmatic/serum level of LPB have been an indirect biomarker of endotoxin leakage from the gut, applied to track factors that interact with the gut inflammation and integrity [53,54] as infections [55,56], heat or/and feed restriction stress [57,58,59,60,61,62,63,64], diet, and bacterial gut composition [65,66,67,68,69,70].

Calprotectin is a calcium-bound protein complex expressed and released by neutrophils in inflamed sites, but also by monocytes, activated macrophages, and dendritic cells [71,72,73,74]. As part of the innate immune response, this protein sequestrates zinc and manganese thus disrupting bacterial and fungal metabolic pathways and exposing these microorganisms to stress [75,76,77]. Other calprotectin functions include chemotaxis and endothelial adhesion of neutrophils, and the upregulation of certain cytokines, as IL-1β, IL-6 and TNF-α [73]. Fecal and circulating levels of calprotectin have been used as a biomarker to track the gut inflammatory activity in human [78,79,80,81,82,83] and canine [74,84,85,86] patients with chronic enteropathies. In swine, the biomarker has been applied to evaluate the intestinal disruption by pathogenic enterobacteria and their toxins [87,88,89,90,91,92], but also to evaluate the positive effects of dietary supplementations on the intestinal inflammation and integrity [92,93,94]. Studies in human health show that the blood levels of calprotectin increase in response to diverse conditions involving tissue damage and inflammation, not exclusively in the intestine [72,95]. Consequently, measuring the calprotectin levels in the feces should provide safer inferences between the biomarker and an intestinal inflammatory process [96].

Lipocalin-2 or Neutrophil-gelatinase associated lipocalin (NGAL) is a glycoprotein highly abundant in the cytoplasm of neutrophils, where it is stored in granules. The NGAL antibacterial activity relies on binding to bacterial siderophores, thus preventing microorganisms from capturing necessary iron for their metabolism [97,98,99]. While increased NGAL levels in the blood and urine have been associated with acute kidney injury [100,101], the upregulation of NGAL expression during intestinal injury and inflammation [102,103] has made the NGAL concentration in the feces a potential biomarker of intestinal impairment in IBD patients [104,105,106,107,108,109]. The same concept has been applied in swine, where fecal lipocalin has been targeted to evaluate the intestinal integrity response to variables such as dietary supplementation [110,111,112], pregnancy, lactation, and obesity in sows [113,114].

Lactoferrin is a glycoprotein secreted by neutrophils and epithelial cells and with affinity for iron, a feature that allows lactoferrin to limit the bacterial growth by reducing the Fe3+ availability for their metabolism. Besides its bacteriostatic potential, lactoferrin also displays direct antibacterial effects and can modulate the immune response [115,116]. Lactoferrin is an important secretion of neutrophils in inflamed sites, thus allowing its fecal concentration to be applied as a biomarker of intestinal inflammatory activity in human patients with chronic enteropathies [117,118,119]. We did not find any report of fecal or circulating lactoferrin as a biomarker in swine, [NO_PRINTED_FORM] thus requiring preliminary studies to understand its applicability in pigs.

Myeloperoxidase (MPO) is an enzyme abundantly found in cytoplasmatic granules of myeloid cells, as neutrophils. In an inflamed site, elicited leucocytes (i) degranulate the enzyme in the extracellular microenvironment and (ii) phagocytizes invading elements, fusing the phagosome with MPO-filled granules. The enzyme reacts with hydrogen peroxide to form hypochlorous acid, a potent bactericidal agent that contributes to the elimination of pathogens [120,121,122]. Molecules of MPO secreted in the intestinal lumen and inside leucocytes debris during inflammatory episodes can be quantified in the feces, thus unveiling an ongoing intestine inflammation. This principle is applied to track enteropathies in humans and dogs [123,124,125], but little was found in swine, where the biomarker did not respond to a chemical challenge to induce enteritis [126]. Studies have related MPO expression and activity to the development of cardiovascular and other inflammatory diseases [127,128,129]. Therefore, the fecal measurement of the biomarker would be recommended to track the intestinal impairment specifically.

Neopterin is a substance produced by activated monocytes/macrophages, with IFN-γ as its major inducer. Neopterin acts in the cellular redox system of leucocytes, promoting the synthesis of oxygen and nitrogen reactive species and boosting their antimicrobial effect [130,131,132]. Serum, urinary and fecal neopterin concentrations have satisfactorily related to chronic enteropathies in humans, with increased values in patients with Crohn’s disease and ulcerative colitis due to its synthesis in inflamed sites [133,134]. Studies in swine have targeted serum neopterin to assess the immune activation during surgeries [135,136] and kidney injury [137], but no study was found applying it as a marker of intestinal injury. Although blood levels of neopterin have been associated with intestinal inflammation, as mentioned above, studies demonstrate that increased circulating concentrations of neopterin can be also related to cardiovascular diseases, cancer, and sepsis in humans [138,139,140,141,142]. This indicates that feces would be the proper material to measure the biomarker when aiming to assess intestinal health.

Intestinal Fatty Acid Binding Protein (I-FABP) is present in the cytoplasm of mature enterocytes, where it binds non-covalent and reversibly to fatty acids thus enhancing their solubility and improving their transportation throughout the cytosol [143,144]. Variants are expressed in many organs and, as intracellular proteins, their presence in the blood stream indicates tissue damage with the leak of protein from harmed cells [145,146,147]. Factors inducing intestinal inflammation and injury as sepsis, ischemia, infection, and chemical challenge have increased the I-FABP serum concentration in varied species, including swine [126,148,149,150,151,152,153,154,155].

Diamine oxidase (DAO) is an intracellular enzyme highly expressed in mature enterocytes (thus present in the tip of intestinal villi) and that catalyzes the oxidation of polyamines, as histamine [156,157,158]. Injuries to the enteric mucosa are related to higher circulating levels of DAO, which leak from the damaged enterocytes into the blood stream [159,160]. Factors compromising the gut integrity as heat stress [161], bacterial [162,163,164,165], viral [166,167] or chemical [126] enteric challenge, ischemia [160,168], and exposure to mycotoxins [163,169] have increased the DAO circulating concentrations, thus confirming its potential to express the mucosa impairment.

Citrulline is an amino acid produced and exclusively released by enterocytes, mainly in the small intestine, through the metabolization of dietary glutamine and its derivatives [170,171]. Decreased circulating levels of citrulline may imply that enterocytes are being damaged, thus compromising the amino acid synthesis and reducing its release in the blood stream. Such a relation is explored in human health to assess the cytotoxic effects of chemo- and radiotherapy on the gut mucosa, which result in mucositis [172,173,174,175]. Citrulline circulating levels have also responded to intestinal cytotoxicity in dogs and rats, but this is still controversial in these species [176,177,178,179]. In swine, the concentration in plasma was reduced in animals with chemically induced enteritis [126], but no other report was found to insure the reliability of citrulline as a biomarker of intestinal damage in pigs.

Zonulin is an important modulator of the tight junctions (TJ), increasing the paracellular permeability to, theoretically, enhance the water outflux and “wash” the luminal surface from harmful elements [180,181]. Its expression in the gut mucosa was shown to be upregulated by bacterial infections [182] and exposure to gluten [183], thus being associated with enteric inflammation. The reaction cascade evoked by zonulin culminates in the displacement of certain TJ proteins, including the zonula occludens-1 (ZO-1). As an evident target of zonulin, ZO-1 is found rearranged in the cytoplasm instead of near the cell membrane, where it mediates the bridge between the external claudin and occludin to the internal cytoskeleton [182,184]. In human health, fecal zonulin is a potential biomarker of Crohn’s Disease activity [185,186], being also applied in studies with dogs to evaluate gut integrity [187,188,189,190]. Serum levels of zonulin have been targeted for the same purpose in swine [111,169,191], although the zonulin expression by other tissues may not allow a secure relation to gut health [192].

Endotoxin is an alternative to host-based biomarkers, being originated from the enteric bacterial community. The term “endotoxin” refers to Lipid A, a glucosamine-based phospholipid that constitutes the hydrophobic portion of LPS from the outer membrane of gram-negative bacteria [193,194]. Given that these bacterial structures are not supposed to reach the blood stream in the absence of factors increasing the intestinal permeability, researchers have already targeted the plasma/serum levels of endotoxin to track the effects of intestinal infection, obesity, gestation, lactation, heat stress and exposure to mycotoxins, and treatments on the inflammatory status of the intestine in varied animal species, including swine [163,169,191,195,196,197,198,199].

D-lactate is the d-isomer of 2-hydroxypropanoate. Differently from its L-isomer, D-lactate is only produced in nanomolar concentrations by mammalian cells, being predominantly originated from the microbial fermentation in the colon [200,201]. Serum d-lactate levels have been purposed as a biomarker of gut injury induced by ischemia, including in swine, since this bacterial byproduct leaks from the gut lumen when the mucosa is compromised [60,155,202,203,204]. Moreover, the biomarker has also been associated with the expression of improvements in the gut health of unchallenged pigs treated with phytobiotic products [110,191] or prebiotic [205]. Challenging factor such as infection by enterotoxigenic *E*. *coli* [196,206], exposure to mycotoxin [207], weaning period [208], and septic shock [152] had their gut damaging effect represented by increased circulating levels of D-lactate in swine.

The measurement of fecal cytokines is an alternative to gene expression assays that require invasive samplings, besides allowing to target their production specifically in the gut mucosa. Studies have reported increased fecal concentrations of cytokines such as IL-6 and IL-8 in humans and dogs with intestinal inflammation [209,210,211], but we did not identify fecal assess of cytokines in swine. Additionally, we do not approach the quantification of cytokines in blood in this review since their circulating levels are not specific for intestinal inflammation. Therefore, inferences about gut health based on the blood measurement of cytokines as a biomarker would be limited without an additional tool to diagnose an enteric inflammatory process.

## 7. Metabolic Biomarkers of Low-Grade, Chronic Intestinal Inflammation

The process of inflammation promotes metabolic reprogramming in a process now known as immunometabolism [212]. Immunometabolism can be further separate into two categories: cellular and tissue. Cellular immunometabolism is the reprogramming of immune cells that regulates cell phenotype and function and environmental conditions and Tissue immunometabolism which is the study of the influence of the immune cells and their metabolic reprogramming on organ metabolism [212]. The main functional feature of metabolic reprogramming under inflammation is the (a) shift in energy production from oxidative phosphorylation to aerobic glycolysis (Warburg effect) and (b) the alteration in epigenetic programming through DNA accessibility and chromatin structure (enhanced histone acetylation and suppressed DNA methylation) [213,214]. What stands out is that immunometabolism provides not only energy and intermediate precursors of macromolecule biosynthesis for cellular and tissue housekeeping functions, but also delivers immune metabolites that serve as essential signaling molecules involved in the immunoregulatory processes that drive immune and inflammatory activation [215,216,217]. The reprogramming of metabolic pathways such as glycolysis, the Krebs cycle, mitochondrial respiration (oxidative phosphorylation) and the pentose phosphate pathway, during immune cell activation all result in the production of these immune metabolites [217] or metabolic regulators [218]. Functionally, immunometabolites to act as signaling effector molecules that modulate transcription factors, alter protein function, epigenetic and posttranslational modifiers, and inhibitors/activators of specific enzyme pathways [217,219,220,221,222,223,224].

### 7.1. Transcription Factors as Metabolic Biomarkers

Hypoxia-inducible factor-1α (HIF1α) is essential to the cellular metabolism and adaptation to cellular stress caused by hypoxia and fundamental reprogrammer of inflammatory cell metabolism that promotes inflammatory gene expression. A set of “signature” anabolic genes/proteins involved in the control of cellular metabolism have their expression regulated by HIF-1α. These proteins include all enzymes in the glycolysis pathway, glucose transporters, transferrin receptor, and the induction of pro-inflammatory genes [225,226]. Further, HIF-1α play a role in effector functions of macrophages and T cells during inflammation.

Peroxisome proliferator-activated receptor gamma (PPAR-γ) is involved sustaining fatty acid metabolism by regulating fatty acid storage and glucose metabolism. The receptor is an endogenous regulator of intestinal inflammation whose levels are suppressed during chronic inflammation [227,228].

Nuclear factor-κ–light-chain-enhancer of activated B cells (NF-κB) regulates multiple components of innate and adaptive immune functions and functions as a focal facilitator of inflammatory responses including inducing the expression of various pro-inflammatory genes, including those encoding cytokines and chemokines. Further, NF-κB is a regulator of energy metabolism networks by controlling the balance between the utilization of glycolysis and mitochondrial respiration [229,230].

### 7.2. Metabolic Intermediates as Metabolic Biomarkers

Succinate is an immunometabolite intermediate of the Kreb’s cycle that plays a crucial role in adenosine triphosphate (ATP) generation in mitochondria but has been found to play a regulatory role as a signaling molecule during intestinal inflammation [231,232]. Specifically, succinate stabilizes HIF-1α in activated macrophages, activates dendritic cells, and post-translationally modifies pro-inflammatory cytokines.

Citrate, like succinate, is an intermediate of the Kreb’s cycle which regulates carbohydrate and lipid metabolism and can have both pro- and anti-inflammatory functions in macrophages and dendritic cells depending on the cellular local of the immunometabolite [233]. Excess citrate, during the Kreb’s cycle, is transported out of the mitochondria into the cytosol of macrophages where it is involved in the production of prostaglandins and nitric oxide, and via the generation of malonyl-CoA and acetyl-CoA impact cytokine production. Citrate can also generate itaconate via aconitate, which promotes an anti-inflammatory response.

Nicotinamide adenine dinucleotide (NAD+) is a critical metabolic intermediate that participates as enzymatic cofactor in redox reactions and as a co-substrate for some enzymes such as sirtuins, adenosine diphosphate ribose transferases and synthases. With these roles, NAD+ metabolism regulates a broad spectrum of cellular functions such as energy metabolism, DNA repair, regulation of the epigenetic landscape and inflammation [234].

Nicotinamide phosphoribosyltransferase (NAMPT) catalyzes the rate-limiting step of the NAD salvage pathway [235] and is ubiquitously detected in all tissues. Extracellular NAMPT, also known as visfatin or pre-B cell colony-enhancing factor (PBEF), has been considered to be a circulating hormone/cytokine released by several tissues has been shown to play a key role metabolism and inflammation [236]. Sirtuins (SIRT) are NAD+ -dependent epigenetic and metabolic regulators [237]. Members of the sirtuin family protect tissue homeostasis by sensing bioenergy demands and react by making alterations in the cell nutrients. Sirtuins play a critical role in homeostasis by stimulating anabolic glycolysis during inflammation and, alternatively, reprogram metabolism by inducing lipolysis/fatty acid oxidation after the removal of the inflammatory trigger.

### 7.3. Post-Translational Modifiers as Metabolic Biomarkers

The kinases AMPK and mTOR mediate signaling pathways that play crucial and opposing functions in immunometabolism of inflammation [238].

AMP-activated protein kinase (AMPK) is a serine threonine kinase that is highly conserved through evolution. This protein is a sensor of cellular energy status found in eukaryotic cells that is activated under conditions of low intracellular ATP. The AMPK is a vital metabolic energy regulator in avian species [239] whose main role is monitoring the ratio of AMP:ATP and to adjust metabolic processes accordingly. Furthermore, AMPK can receive signals of cellular energy state and respond, via phosphorylation, that then influences glycolysis/gluconeogenesis, protein synthesis, fatty acid synthesis, and fatty acid oxidation. Numerous studies have shown that the phosphorylation of AMPK hampers inflammatory responses, whereas dephosphorylation of AMPK is associated with increased inflammation [240,241,242]. The reprogramming of cellular metabolism is imperative for immune cells responding to environmental cues and AMPK directs the metabolic adaptation of immune cells upon nutrient limitation and contributes to the immune response in vivo [242]. Lastly, AMPK modifies inflammation by antagonizing NF-κB, transcription of pro-inflammatory cytokines [240]. Therefore, AMPK would be identified is a negative immunometabolic regulator of inflammation.

Mechanistic target of rapamycin (mTOR) is a serine/threonine kinase that also plays a role in cell growth and metabolism by sensing environmental cues. This kinase is phosphorylated when nutrients are in abundance especially when immune cells are in metabolically demanding situations, such as stimulation with growth factors, nutrient availability, and immune regulatory signals [243,244]. Phosphorylated mTOR senses cues from the immune microenvironment and elevates immune cell growth and proliferation [245]. The mTOR pathway aids in a cell’s ability to meet high metabolic demands by promoting anabolic process, such as lipid and protein synthesis and repressing catabolic processes [246]. Moreover, mTOR is considered a positive immunometabolic regulator of inflammation.

## 8. Conclusions

Maintenance of intestinal health is critical to a successful animal production industry. For decades the swine and poultry industry has relied upon the inclusion of AGPs in the feed to achieve this goal and by extension improve growth performance. With the removal of AGPs, the emergence of chronic, low-level gut inflammation has come to the forefront of concern. Although the mechanisms by which AGPs promote intestinal health and suppress the development of inflammation are not well understood, it is generally believed that it occurs as a result of stabilization of the microbial communities within the gut as well as suppression of pathogen colonization.

With the removal of AGPs from feed, there is now a critical need for examining new strategies that the animal industry can utilize to control the development of chronic low-grade inflammation. The intestine of swine is constantly exposed to a number of environmental triggers including weaning period, intestinal pathogens, poor quality of feed ingredients, high energy diets, changes in feed formulation, and intrinsic chemical and physical characteristics of dietary ingredients that stimulate inflammation and lead to a reduction in performance. Additionally, other conditions such as pregnancy, lactation and obesity have also been associated with alterations in the intestinal homeostasis.

The chronic low-grade inflammatory response that occur in the intestine results in impairments to the digestive function and, consequently, decreased growth rate. Besides capturing resources from functions that are essential to the animal productivity, pro-inflammatory mediators promote muscle catabolism to supply amino acids and energy substrates for the inflammatory process. In addition to the detrimental effects on performance, the compromising of carcass traits can be related to chronic inflammation and the consequent shifts in the systemic metabolism.

Intestinal low-grade, chronic inflammation is a key contributor to complications affecting production animal performance. To support novel approaches to these complications, it is vital to expand our understanding of the inflammatory dynamics and to monitor them. Here we summarize potential biomarkers reflecting intestinal-specific inflammatory dynamics for use in swine and poultry production worldwide. Immune responses have been linked to energy metabolism (immunometabolism); thus, the integration of these systems and their cooperation in responding to fluctuations in the energy and nutritional environment would be beneficial. The identification of immunometabolic biomarkers should provide dynamic and definitive predictors and biomarkers of low, grade chronic inflammation, but also provide indicators of successful nutritional or feed additive intervention strategies.

## Data Availability

Not applicable.

## References

[1] Choct M. (2009). Managing Gut Health through Nutrition. Br. Poult. Sci..

[2] Svihus B. (2014). Function of the Digestive System. J. Appl. Poult. Res..

[3] Celi P., Verlhac V., Pérez Calvo E., Schmeisser J., Kluenter A.M. (2019). Biomarkers of Gastrointestinal Functionality in Animal Nutrition and Health. Anim. Feed Sci. Technol..

[4] Liu H.Y., Dicksved J., Rakhshandeh A., Cai D. (2021). Integrated Role of Nutrition and Digestive Physiology for Animal Health. Front. Vet. Sci..

[5] Abraham C., Medzhitov R. (2011). Interactions between the Host Innate Immune System and Microbes in Inflammatory Bowel Disease. Gastroenterology.

[6] Nicholson J.K., Holmes E., Kinross J., Burcelin R., Gibson G., Jia W., Pettersson S. (2012). Host-Gut Microbiota Metabolic Interactions. Science (1979).

[7] Kinnebrew M.A., Pamer E.G. (2012). Innate Immune Signaling in Defense against Intestinal Microbes. Immunol. Rev..

[8] Alexander M., Turnbaugh P.J. (2020). Deconstructing Mechanisms of Diet-Microbiome-Immune Interactions. Immunity.

[9] Kogut M.H. (2019). The Effect of Microbiome Modulation on the Intestinal Health of Poultry. Anim. Feed Sci. Technol..

[10] Nobs S.P., Zmora N., Elinav E. (2020). Nutrition Regulates Innate Immunity in Health and Disease. Annu. Rev. Nutr..

[11] Kogut M.H., Arsenault R.J. (2015). A Role for the Non-Canonical Wnt-β-Catenin and TGF-β Signaling Pathways in the Induction of Tolerance during the Establishment of a Salmonella Enterica Serovar Enteritidis Persistent Cecal Infection in Chickens. Front. Vet. Sci..

[12] Lee M.D., Ipharraguerre I.R., Arsenault R.J., Lyte M., Lyte J.M., Humphrey B., Angel R., Korver D.R. (2022). Informal Nutrition Symposium: Leveraging the Microbiome (and the Metabolome) for Poultry Production. Poult. Sci..

[13] Makowski L., Chaib M., Rathmell J.C. (2020). Immunometabolism: From Basic Mechanisms to Translation. Immunol. Rev..

[14] Lee Y.S., Wollam J., Olefsky J.M. (2018). An Integrated View of Immunometabolism. Cell.

[15] Michaudel C., Sokol H. (2020). The Gut Microbiota at the Service of Immunometabolism. Cell Metab..

[16] Goo D., Kim J.H., Choi H.S., Park G.H., Han G.P., Kil D.Y. (2019). Effect of Stocking Density and Sex on Growth Performance, Meat Quality, and Intestinal Barrier Function in Broiler Chickens. Poult. Sci..

[17] Reisinger N., Emsenhuber C., Doupovec B., Mayer E., Schatzmayr G., Nagl V., Grenier B. (2020). Endotoxin Translocation and Gut Inflammation Are Increased in Broiler Chickens Receiving an Oral Lipopolysaccharide (LPS) Bolus during Heat Stress. Toxins.

[18] Goo D., Kim J.H., Park G.H., Reyes J.B.D., Kil D.Y. (2019). Effect of Heat Stress and Stocking Density on Growth Performance, Breast Meat Quality, and Intestinal Barrier Function in Broiler Chickens. Animals.

[19] Goossens E., Debyser G., Callens C., de Gussem M., Dedeurwaerder A., Devreese B., Haesebrouck F., Flügel M., Pelzer S., Thiemann F. (2018). Elevated Faecal Ovotransferrin Concentrations Are Indicative for Intestinal Barrier Failure in Broiler Chickens. Vet. Res..

[20] Lee K.A., Roth R.A., LaPres J.J. (2007). Hypoxia, Drug Therapy and Toxicity. Pharmacol. Ther..

[21] Taylor C.T., Colgan S.P. (2007). Hypoxia and Gastrointestinal Disease. J. Mol. Med..

[22] Oviedo-Rondón E.O. (2019). Holistic View of Intestinal Health in Poultry. Anim. Feed Sci. Technol..

[23] Tabler T.W., Greene E.S., Orlowski S.K., Hiltz J.Z., Anthony N.B., Dridi S. (2020). Intestinal Barrier Integrity in Heat-Stressed Modern Broilers and Their Ancestor Wild Jungle Fowl. Front. Vet. Sci..

[24] Kogut M.H., Genovese K.J., Swaggerty C.L., He H., Broom L. (2018). Inflammatory Phenotypes in the Intestine of Poultry: Not All Inflammation Is Created Equal. Poult. Sci..

[25] Broom L.J., Kogut M.H. (2019). Deciphering Desirable Immune Responses from Disease Models with Resistant and Susceptible Chickens. Poult. Sci..

[26] Lauridsen C. (2019). From Oxidative Stress to Inflammation: Redox Balance and Immune System. Poult. Sci..

[27] Roseth A.G., Aadland E., Jahnsen J., Raknerud N. (1997). Assessment of Disease Activity in Ulcerative Colitis by Faecal Calprotectin, a Novel Granulocyte Marker Protein. Digestion.

[28] Rychlik I., Elsheimer-Matulova M., Kyrova K. (2014). Gene Expression in the Chicken Caecum in Response to Infections with Non-Typhoid Salmonella. Vet. Res..

[29] He B., Bortoluzzi C., King W.D., Graugnard D., Dawson K.A., Applegate T.J. (2019). Zinc Source Influences the Gene Expression of Zinc Transporters in Jejunum and Cecal Tonsils during Broiler Challenge with Eimeria Maxima and Clostridium Perfringens. Poult. Sci..

[30] Bento A.F., Leite D.F.P., Marcon R., Claudino R.F., Dutra R.C., Cola M., Martini A.C., Calixto J.B. (2012). Evaluation of Chemical Mediators and Cellular Response during Acute and Chronic Gut Inflammatory Response Induced by Dextran Sodium Sulfate in Mice. Biochem. Pharmacol..

[31] Lochmiller R.L., Deerenberg C. (2000). Trade-Offs in Evolutionary Immunology: Just What Is the Cost of Immunity?. Oikos.

[32] Corzo A., Kidd M.T., Dozier W.A., Pharr G.T., Koutsos E.A. (2007). Dietary Threonine Needs for Growth and Immunity of Broilers Raised under Different Litter Conditions. J. Appl. Poult. Res..

[33] Jiang Z., Schatzmayr G., Mohnl M., Applegate T.J. (2010). Net Effect of an Acute Phase Response-Partial Alleviation with Probiotic Supplementation. Poult. Sci..

[34] Klasing K.C., Laurin D.E., Peng R.K., Fry D.M. (1987). Immunologically Mediated Growth Depression in Chicks: Influence of Feed Intake, Corticosterone and Interleukin-1. J. Nutr..

[35] Medzhitov R. (2021). The Spectrum of Inflammatory Responses. Science (1979).

[36] Dal Pont G.C., Belote B.L., Lee A., Bortoluzzi C., Eyng C., Sevastiyanova M., Khadem A., Santin E., Farnell Y.Z., Gougoulias C. (2021). Novel Models for Chronic Intestinal Inflammation in Chickens: Intestinal Inflammation Pattern and Biomarkers. Front. Immunol..

[37] Caspary W.F. (1992). Physiology and Pathophysiology of Intestinal Absorption. Am. J. Clin. Nutr..

[38] Awad W.A., Ghareeb K., Abdel-Raheem S., Böhm J. (2009). Effects of Dietary Inclusion of Probiotic and Synbiotic on Growth Performance, Organ Weights, and Intestinal Histomorphology of Broiler Chickens. Poult. Sci..

[39] Kraieski A.L., Hayashi R.M., Sanches A., Almeida G.C., Santin E. (2017). Effect of Aflatoxin Experimental Ingestion and Eimeira Vaccine Challenges on Intestinal Histopathology and Immune Cellular Dynamic of Broilers: Applying an Intestinal Health Index. Poult. Sci..

[40] Belote B.L., Tujimoto-Silva A., Hümmelgen P.H., Sanches A.W.D., Wammes J.C.S., Hayashi R.M., Santin E. (2018). Histological Parameters to Evaluate Intestinal Health on Broilers Challenged with Eimeria and C Lostridium Perfringens with or without Enramycin as Growth Promoter. Poult. Sci..

[41] Belote B.L., Soares I., Tujimoto-Silva A., Sanches A.W.D., Kraieski A.L., Santin E. (2019). Applying I See inside Histological Methodology to Evaluate Gut Health in Broilers Challenged with Eimeria. Vet. Parasitol. X.

[42] Belote B.L., Soares I., Tujimoto-Silva A., Tirado A.G.C., Martins C.M., Carvalho B., Gonzalez-Esquerra R., Rangel L.F.S., Santin E. (2021). Field Evaluation of Feeding Spray-Dried Plasma in the Starter Period on Final Performance and Overall Health of Broilers. Poult. Sci..

[43] Sanches A.W.D., Belote B.L., Hümmelgen P., Heemann A.C.W., Soares I., Tujimoto-Silva A., Tirado A.G.C., Cunha A.F., Santin E. (2020). Basal and Infectious Enteritis in Broilers Under the I See Inside Methodology: A Chronological Evaluation. Front. Vet. Sci..

[44] Santin E., Carlos Paulillo A., Maiorka A., Okada Nakaghi L.S., Macari M., da Silva A.V.F., Carlos Alessi A. (2003). Evaluation of the Efficacy of Saccharomyces Cerevisiae Cell Wall to Ameliorate the Toxic Effects of Aflatoxin in Broilers. Int. J. Poult. Sci..

[45] Ortatatli M., Oǧuz H., Hatipoǧlu F., Karaman M. (2005). Evaluation of Pathological Changes in Broilers during Chronic Aflatoxin (50 and 100 Ppb) and Clinoptilolite Exposure. Res. Vet. Sci..

[46] Ducatelle R., Goossens E., de Meyer F., Eeckhaut V., Antonissen G., Haesebrouck F., van Immerseel F. (2018). Biomarkers for Monitoring Intestinal Health in Poultry: Present Status and Future Perspectives. Vet. Res..

[47] Wan Y., Freeswick P.D., Khemlani L.S., Kispert P.H., Wang S.C., Su G.L., Billiar T.R. (1995). Role of Lipopolysaccharide (LPS), Interleukin-1, Interleukin-6, Tumor Necrosis Factor, and Dexamethasone in Regulation of LPS-Binding Protein Expression in Normal Hepatocytes and Hepatocytes from LPS-Treated Rats. Infect. Immun..

[48] Read T.E., Harris H.W., Grunfeld C., Feingold K.R., Calhoun M.C., Kane J.P., Rapp’ J.H. (1993). Chylomicrons Enhance Endotoxin Excretion in Bile. Infect. Immun..

[49] Berbee J.F.P., Havekes L.M., Rensen P.C.N. (2005). Apolipoproteins Modulate the Inflammatory Response to Lipopolysaccharide. J. Endotoxin Res..

[50] Krasity B.C., Troll J.V., Weiss J.P., McFall-Ngai M.J. (2011). LBP/BPI Proteins and Their Relatives: Conservation over Evolution and Roles in Mutualism. Biochem. Soc. Trans..

[51] González R., Brokordt K., Rojas R., Schmitt P. (2020). Molecular Characterization and Expression Patterns of Two LPS Binding /Bactericidal Permeability-Increasing Proteins (LBP/BPIs) from the Scallop Argopecten Purpuratus. Fish Shellfish Immunol..

[52] Weiss J. (2003). Bactericidal/Permeability-Increasing Protein (BPI) and Lipopolysaccharide-Binding Protein (LBP): Structure, Function and Regulation in Host Defence against Gram-Negative Bacteria. Biochem. Soc. Trans..

[53] Roberts L.M., Buford T.W. (2021). Lipopolysaccharide Binding Protein Is Associated with CVD Risk in Older Adults. Aging Clin. Exp. Res..

[54] Kvidera S.K., Dickson M.J., Abuajamieh M., Snider D.B., Fernandez M.V.S., Johnson J.S., Keating A.F., Gorden P.J., Green H.B., Schoenberg K.M. (2017). Intentionally Induced Intestinal Barrier Dysfunction Causes Inflammation, Affects Metabolism, and Reduces Productivity in Lactating Holstein Cows. J. Dairy Sci..

[55] Oliva A., Aversano L., de Angelis M., Mascellino M.T., Miele M.C., Morelli S., Battaglia R., Iera J., Bruno G., Corazziari E.S. (2020). Persistent Systemic Microbial Translocation, Inflammation, and Intestinal Damage during Clostridioides Difficile Infection. Open Forum. Infect. Dis..

[56] Zhang M.X., Song T.Z., Zheng H.Y., Wang X.H., Lu Y., Zhang H.D., Li T., Pang W., Zheng Y.T. (2019). Superior Intestinal Integrity and Limited Microbial Translocation Are Associated with Lower Immune Activation in SIVmac239-Infected Northern Pig-Tailed Macaques (*Macaca Leonina*). Zool. Res..

[57] Cui Y., Wang C., Hao Y., Gu X., Wang H. (2019). Chronic Heat Stress Induces Acute Phase Responses and Serum Metabolome Changes in Finishing Pigs. Animals.

[58] Mayorga E.J., Kvidera S.K., Horst E.A., Al-Qaisi M., Dickson M.J., Seibert J.T., Lei S., Keating A.F., Ross J.W., Rhoads R.P. (2018). Effects of Zinc Amino Acid Complex on Biomarkers of Gut Integrity and Metabolism during and Following Heat Stress or Feed Restriction in Pigs. J. Anim. Sci..

[59] Abuajamieh M., Kvidera S.K., Mayorga E.J., Kaiser A., Lei S., Seibert J.T., Horst E.A., Fernandez M.V.S., Ross J.W., Selsby J.T. (2018). The Effect of Recovery from Heat Stress on Circulating Bioenergetics and Inflammatory Biomarkers. J. Anim. Sci..

[60] Sanz Fernandez M.V., Pearce S.C., Mani V., Gabler N.K., Metzger L., Patience J.F., Rhoads R.P., Baumgard L.H. (2014). Effects of Dairy Products on Intestinal Integrity in Heat-Stressed Pigs. Temperature.

[61] Opgenorth J., Abuajamieh M., Horst E.A., Kvidera S.K., Johnson J.S., Mayorga E.J., Sanz-Fernandez M.V., Al-Qaisi M.A., DeFrain J.M., Kleinschmit D.H. (2021). The Effects of Zinc Amino Acid Complex on Biomarkers of Gut Integrity, Inflammation, and Metabolism in Heat-Stressed Ruminants. J. Dairy Sci..

[62] Kvidera S.K., Horst E.A., Sanz Fernandez M.V., Abuajamieh M., Ganesan S., Gorden P.J., Green H.B., Schoenberg K.M., Trout W.E., Keating A.F. (2017). Characterizing Effects of Feed Restriction and Glucagon-like Peptide 2 Administration on Biomarkers of Inflammation and Intestinal Morphology. J. Dairy Sci..

[63] Mayorga E.J., Kvidera S.K., Seibert J.T., Horst E.A., Abuajamieh M., Al-Qaisi M., Lei S., Ross J.W., Johnson C.D., Kremer B. (2019). Effects of Dietary Chromium Propionate on Growth Performance, Metabolism, and Immune Biomarkers in Heat-Stressed Finishing Pigs. J. Anim. Sci..

[64] Horst E.A., Mayorga E.J., Al-Qaisi M., Rodriguez-Jimenez S., Goetz B.M., Abeyta M.A., Gorden P.J., Kvidera S.K., Baumgard L.H. (2020). Evaluating Effects of Zinc Hydroxychloride on Biomarkers of Inflammation and Intestinal Integrity during Feed Restriction. J. Dairy Sci..

[65] Mayorga E.J., Horst E.A., Al-Qaisi M., Goetz B.M., Abeyta M.A., Rodríguez-Jiménez S., Lei S., Acosta J.A., Patience J.F., Serao M.R. (2021). Effects of Continuously Infusing Glucose or Casein into the Terminal Ileum on Biomarkers of Metabolism, Inflammation, and Intestinal Morphology in Growing Pigs 1. J. Anim. Sci..

[66] Zhu Y., Lin X., Zhao F., Shi X., Li H., Li Y., Zhu W., Xu X., Lu C., Zhou G. (2015). Meat, Dairy and Plant Proteins Alter Bacterial Composition of Rat Gut Bacteria. Sci. Rep..

[67] Petry A.L., Huntley N.F., Bedford M.R., Patience J.F. (2020). Xylanase Increased the Energetic Contribution of Fiber and Improved the Oxidative Status, Gut Barrier Integrity, and Growth Performance of Growing Pigs Fed Insoluble Corn-Based Fiber 1. J. Anim. Sci..

[68] Bischoff S.C., Kaden-Volynets V., Filipe Rosa L., Guseva D., Seethaler B. (2021). Regulation of the Gut Barrier by Carbohydrates from Diet–Underlying Mechanisms and Possible Clinical Implications. Int. J. Med. Microbiol..

[69] Ahmad M.I., Zou X., Ijaz M.U., Hussain M., Liu C., Xu X., Zhou G., Li C. (2019). Processed Meat Protein Promoted Inflammation and Hepatic Lipogenesis by Upregulating Nrf2/Keap1 Signaling Pathway in Glrx-Deficient Mice. J. Agric. Food Chem..

[70] Panasevich M.R., Meers G.M., Linden M.A., Booth X.F.W., Perfield Ii J.W., Fritsche K.L., Wankhade U.D., Chintapalli S.V., Shankar K., Ibdah J.A. (2018). High-Fat, High-Fructose, High-Cholesterol Feeding Causes Severe NASH and Cecal Microbiota Dysbiosis in Juvenile Ossabaw Swine. Am. J. Physiol. Endocrinol. Metab..

[71] Ayling R.M., Kok K. (2018). Fecal Calprotectin. Adv. Clin. Chem..

[72] Roseth A.G., Fagerhol M.K., Aadland E., Schjbnsby H., Rgseth A.G. (1992). Assessment of the Neutrophil Dominating Protein Calprotectin in Feces A Methodologic Study. Scand. J. Gastroenterol..

[73] Jukic A., Bakiri L., Wagner E.F., Tilg H., Adolph T.E. (2021). Calprotectin: From Biomarker to Biological Function. Gut.

[74] Heilmann R.M., Nestler J., Schwarz J., Grützner N., Ambrus A., Seeger J., Suchodolski J.S., Steiner J.M., Gurtner C. (2019). Mucosal Expression of S100A12 (Calgranulin C) and S100A8/A9 (Calprotectin) and Correlation with Serum and Fecal Concentrations in Dogs with Chronic Inflammatory Enteropathy. Vet. Immunol. Immunopathol..

[75] Kehl-Fie T.E., Chitayat S., Hood M.I., Damo S., Restrepo N., Garcia C., Munro K.A., Chazin W.J., Skaar E.P. (2011). Nutrient Metal Sequestration by Calprotectin Inhibits Bacterial Superoxide Defense, Enhancing Neutrophil Killing of *Staphylococcus aureus*. Cell Host Microbe.

[76] Nakashige T.G., Zygiel E.M., Drennan C.L., Nolan E.M. (2017). Nickel Sequestration by the Host-Defense Protein Human Calprotectin. J. Am. Chem. Soc..

[77] Burcham L.R., le Breton Y., Radin J.N., Spencer B.L., Deng L., Hiron A., Ransom M.R., Mendonça J.d.C., Belew A.T., El-Sayed N.M. (2020). Identification of Zinc-Dependent Mechanisms Used by Group b Streptococcus to Overcome Calprotectin-Mediated Stress. mBio.

[78] Schoepfer A.M., Trummler M., Seeholzer P., Seibold-Schmid B., Seibold F. (2008). Discriminating IBD from IBS: Comparison of the Test Performance of Fecal Markers, Blood Leukocytes, CRP, and IBD Antibodies. Inflamm. Bowel Dis..

[79] Jusué V., Chaparro M., Gisbert J.P. (2018). Accuracy of Fecal Calprotectin for the Prediction of Endoscopic Activity in Patients with Inflammatory Bowel Disease. Dig. Liver Dis..

[80] Mosli M.H., Zou G., Garg S.K., Feagan S.G., MacDonald J.K., Chande N., Sandborn W.J., Feagan B.G. (2015). C-Reactive Protein, Fecal Calprotectin, and Stool Lactoferrin for Detection of Endoscopic Activity in Symptomatic Inflammatory Bowel Disease Patients: A Systematic Review and Meta-Analysis. Am. J. Gastroenterol..

[81] Schoepfer A.M., Beglinger C., Straumann A., Trummler M., Renzulli P., Seibold F. (2009). Ulcerative Colitis: Correlation of the Rachmilewitz Endoscopic Activity Index with Fecal Calprotectin, Clinical Activity, C-Reactive Protein, and Blood Leukocytes. Inflamm. Bowel Dis..

[82] Tibble J., Teahon K., Thjodleifsson B., Roseth A., Sigthorsson G., Bridger S., Foster R., Sherwood R., Fagerhol M., Bjarnason I. (2000). A Simple Method for Assessing Intestinal Inflammation in Crohn’s Disease. Gut.

[83] Carroccio A., Iacono G., Cottone M., di Prima L., Cartabellotta F., Cavataio F., Scalici C., Montalto G., Fede G.D., Rini G. (2003). Diagnostic Accuracy of Fecal Calprotectin Assay in Distinguishing Organic Causes of Chronic Diarrhea from Irritable Bowel Syndrome: A Prospective Study in Adults and Children. Clin. Chem..

[84] Heilmann R.M., Berghoff N., Mansell J., Grützner N., Parnell N.K., Gurtner C., Suchodolski J.S., Steiner J.M. (2018). Association of Fecal Calprotectin Concentrations with Disease Severity, Response to Treatment, and Other Biomarkers in Dogs with Chronic Inflammatory Enteropathies. J. Vet. Intern. Med..

[85] Grellet A., Heilmann R.M., Vet M., Lecoindre P., Feugier A., Day M.J., Peeters D., Freiche V., Hernandez J., Grandjean D. (2013). Fecal Calprotectin Concentrations in Adult Dogs with Chronic Diarrhea. Am. J. Vet. Res..

[86] Heilmann R.M., Jergens A.E., Ackermann M.R., Barr J.W., Suchodolski J.S., Steiner J.M. (2012). Serum Calprotectin Concentrations in Dogs with Idiopathic Inflammatory Bowel Disease. Am. J. Vet. Res..

[87] Barbosa J.A., Rodrigues L.A., Columbus D.A., Aguirre J.C.P., Harding J.C.S., Cantarelli V.S., Costa M.d.O. (2021). Experimental Infectious Challenge in Pigs Leads to Elevated Fecal Calprotectin Levels Following Colitis, but Not Enteritis. Porc. Health Manage..

[88] Boeckman J.X., Sprayberry S., Korn A.M., Suchodolski J.S., Paulk C., Genovese K., Rech R.R., Giaretta P.R., Blick A.K., Callaway T. (2022). Effect of Chronic and Acute Enterotoxigenic *E. coli* Challenge on Growth Performance, Intestinal Inflammation, Microbiome, and Metabolome of Weaned Piglets. Sci. Rep..

[89] Šplíchal I., Fagerhol M.K., Trebichavský I., Šplíchalová A., Schulze J. (2005). The Effect of Intestinal Colonization of Germ-Free Pigs with *Escherichia coli* on Calprotectin Levels in Plasma, Intestinal and Bronchoalveolar Lavages. Immunobiology.

[90] Bogere P., Choi J., Heo J. (2019). Optimization of Fecal Calprotectin Assay for Pig Samples. J. Agric. Life Sci..

[91] Grellet A., Heilmann R.M., Polack B., Feugier A., Boucraut-Baralon C., Grandjean D., Grützner N., Suchodolski J.S., Steiner J.M., Chastant-Maillard S. (2016). Influence of Breed Size, Age, Fecal Quality, and Enteropathogen Shedding on Fecal Calprotectin and Immunoglobulin A Concentrations in Puppies During the Weaning Period. J. Vet. Intern. Med..

[92] Xiao D., Wang Y., Liu G., He J., Qiu W., Hu X., Feng Z., Ran M., Nyachoti C.M., Kim S.W. (2014). Effects of Chitosan on Intestinal Inflammation in Weaned Pigs Challenged by Enterotoxigenic *Escherichia coli*. PLoS ONE.

[93] Slinger K.R., Stewart A.H., Daniel Z.C.T.R., Hall H., Masey O’Neill H.V., Bedford M.R., Parr T., Brameld J.M. (2019). The Association between Faecal Host DNA or Faecal Calprotectin and Feed Efficiency in Pigs Fed Yeast-Enriched Protein Concentrate. Animal.

[94] Mazgaj R., Lipiński P., Szudzik M., Jończy A., Kopeć Z., Stankiewicz A.M., Kamyczek M., Swinkels D., Żelazowska B., Starzyński R.R. (2021). Comparative Evaluation of Sucrosomial Iron and Iron Oxide Nanoparticles as Oral Supplements in Iron Deficiency Anemia in Piglets. Int. J. Mol. Sci..

[95] Kristinsson J., Roseth A., Fagerhol M.K., Aadland E., Schjonsby H., Bormer O.P., Raknerud N., Nygaard K. (1998). Fecal Calprotectin Concentration in Patients with Colorectal Carcinoma. Dis. Colon. Rectum..

[96] Bunn S.K., Bisset W.M., Main M.J.C., Golden B.E. (2001). Fecal Calprotectin as a Measure of Disease Activity in Childhood Inflammatory Bowel Disease. J. Pediatr. Gastroenterol. Nutr..

[97] Goetz D.H., Holmes M.A., Borregaard N., Bluhm M.E., Raymond K.N., Strong R.K. (2002). The Neutrophil Lipocalin NGAL Is a Bacteriostatic Agent That Interferes with Siderophore-Mediated Iron Acquisition Ation, Olfaction, Pheromone Transport, Prostaglandin Synthesis, Modulation of Cell Growth and Metabolism, Regulation of the Immune Response, Tissue Development, and Animal Behavior. However, Some of These Functional Assignments Have Been Made on Very Indirect or Circum. Mol. Cell.

[98] Xiao X., Yeoh B.S., Vijay-Kumar M. (2017). Lipocalin 2: An Emerging Player in Iron Homeostasis and Inflammation. Annu. Rev. Nutr..

[99] Moschen A.R., Adolph T.E., Gerner R.R., Wieser V., Tilg H. (2017). Lipocalin-2: A Master Mediator of Intestinal and Metabolic Inflammation. Trends Endocrinol. Metab..

[100] Li D., Yan Sun W., Fu B., Xu A., Wang Y. (2020). Lipocalin-2—The Myth of Its Expression and Function. Basic Clin. Pharmacol. Toxicol..

[101] Makris K., Rizos D., Kafkas N., Haliassos A. (2012). Neurophil Gelatinase-Associated Lipocalin as a New Biomarker in Laboratory Medicine. Clin. Chem. Lab. Med..

[102] Playford R.J., Belo A., Poulsom R., Fitzgerald A.J., Harris K., Pawluczyk I., Ryon J., Darby T., Nilsen-Hamilton M., Ghosh S. (2006). Effects of Mouse and Human Lipocalin Homologues 24p3/Lcn2 and Neutrophil Gelatinase-Associated Lipocalin on Gastrointestinal Mucosal Integrity and Repair. Gastroenterology.

[103] Raffatellu M., George M.D., Akiyama Y., Hornsby M.J., Nuccio S.P., Paixao T.A., Butler B.P., Chu H., Santos R.L., Berger T. (2009). Lipocalin-2 Resistance Confers an Advantage to Salmonella Enterica Serotype Typhimurium for Growth and Survival in the Inflamed Intestine. Cell Host Microbe.

[104] Zollner A., Schmiderer A., Reider S.J., Oberhuber G., Pfister A., Texler B., Watschinger C., Koch R., Effenberger M., Raine T. (2021). Faecal Biomarkers in Inflammatory Bowel Diseases: Calprotectin versus Lipocalin-2-A Comparative Study. J. Crohns Colitis.

[105] Thorsvik S., Damås J.K., Granlund A.B., Flo T.H., Bergh K., Østvik A.E., Sandvik A.K. (2017). Fecal Neutrophil Gelatinase-Associated Lipocalin as a Biomarker for Inflammatory Bowel Disease. J. Gastroenterol. Hepatol..

[106] Chassaing B., Srinivasan G., Delgado M.A., Young A.N., Gewirtz A.T., Vijay-Kumar M. (2012). Fecal Lipocalin 2, a Sensitive and Broadly Dynamic Non-Invasive Biomarker for Intestinal Inflammation. PLoS ONE.

[107] Nielsen O.H., Gionchetti P., Ainsworth M., Vainer B., Campieri M., Borregaard N., Kjeldsen L. (1999). Rectal Dialysate and Fecal Concentrations of Neutrophil Gelatinase-Associated Lipocalin, Interleukin-8, and Tumor Necrosis Factor-in Ulcerative Colitis. Am. J. Gastroentherology.

[108] Bakke I., Walaas G.A., Bruland T., Røyset E.S., van Beelen Granlund A., Escudero-Hernández C., Thorsvik S., Münch A., Sandvik A.K., Østvik A.E. (2021). Mucosal and Faecal Neutrophil Gelatinase-Associated Lipocalin as Potential Biomarkers for Collagenous Colitis. J. Gastroenterol..

[109] Thorsvik S., Bakke I., van Beelen Granlund A., Røyset E.S., Damås J.K., Østvik A.E., Sandvik A.K. (2018). Expression of Neutrophil Gelatinase-Associated Lipocalin (NGAL) in the Gut in Crohn’s Disease. Cell Tissue Res..

[110] Wang Y., Yang Z., Zhou Y., Tan J., Sun H., Sun D., Mu Y., Peng J., Wei H. (2022). Effects of Different Amino Acid Levels and a Carvacrol–Thymol Blend on Growth Performance and Intestinal Health of Weaned Pigs. J. Anim. Sci. Biotechnol..

[111] Cheng C., Wei H., Xu C., Xie X., Jiang S., Peng J. (2018). Maternal Soluble Fiber Diet during Pregnancy Changes the Intestinal Microbiota, Improves Growth Performance, and Reduces Intestinal Permeability in Piglets. Appl. Environ. Microbiol..

[112] Xu S., Shi J., Dong Y., Li Z., Wu X., Lin Y., Che L., Li J., Feng B., Fang Z. (2020). Fecal Bacteria and Metabolite Responses to Dietary Lysozyme in a Sow Model from Late Gestation until Lactation. Sci. Rep..

[113] Cheng C., Wu X., Zhang X., Zhang X., Peng J. (2020). Obesity of Sows at Late Pregnancy Aggravates Metabolic Disorder of Perinatal Sows and Affects Performance and Intestinal Health of Piglets. Animals.

[114] Cheng C., Wei H., Yu H., Xu C., Jiang S., Peng J. (2018). Metabolic Syndrome during Perinatal Period in Sows and the Link with Gut Microbiota and Metabolites. Front. Microbiol..

[115] Adlerova L., Bartoskova A., Faldyna M. (2008). Lactoferrin: A Review. Vet. Med..

[116] Legrand D., Mazurier J. (2010). A Critical Review of the Roles of Host Lactoferrin in Immunity. BioMetals.

[117] Buderus S., Boone J.H., Lentze M.J. (2015). Fecal Lactoferrin: Reliable Biomarker for Intestinal Inflammation in Pediatric IBD. Gastroenterol. Res. Pract..

[118] Sipponen T. (2013). Diagnostics and Prognostics of Inflammatory Bowel Disease with Fecal Neutrophil-Derived Biomarkers Calprotectin and Lactoferrin. Dig. Dis..

[119] Gisbert J.P., McNicholl A.G., Gomollon F. (2009). Questions and Answers on the Role of Fecal Lactoferrin as a Biological Marker in Inflammatory Bowel Disease. Inflamm. Bowel Dis..

[120] Hansberry D.R., Shah K., Agarwal P., Agarwal N. (2017). Fecal Myeloperoxidase as a Biomarker for Inflammatory Bowel Disease. Cureus.

[121] Huang J., Milton A., Arnold R.D., Huang H., Smith F., Panizzi J.R., Panizzi P. (2016). Methods for Measuring Myeloperoxidase Activity toward Assessing Inhibitor Efficacy in Living Systems. J. Leukoc. Biol..

[122] Klebanoff S.J., Kettle A.J., Rosen H., Winterbourn C.C., Nauseef W.M. (2013). Myeloperoxidase: A Front-Line Defender against Phagocytosed Microorganisms. J. Leukoc. Biol..

[123] Masoodi I., Kochhar R., Dutta U., Vaishnavi C., Prasad K.K., Vaiphei K., Kaur S., Singh K. (2009). Fecal Lactoferrin, Myeloperoxidase and Serum C-Reactive Are Effective Biomarkers in the Assessment of Disease Activity and Severity in Patients with Idiopathic Ulcerative Colitis. J. Gastroenterol. Hepatol..

[124] Hanifeh M., Sankari S., Rajamäki M.M., Syrjä P., Kilpinen S., Suchodolski J.S., Heilmann R.M., Guadiano P., Lidbury J., Steiner J.M. (2018). S100A12 Concentrations and Myeloperoxidase Activities Are Increased in the Intestinal Mucosa of Dogs with Chronic Enteropathies. BMC Vet. Res..

[125] Masoodi I., Dutta U., Vaiphei K., Kochhar R., Vaishnavi C., Hussain S., Prasad K.K., Singh K. (2012). Evaluation of Fecal Myeloperoxidase as a Biomarker of Disease Activity and Severity in Ulcerative Colitis. Dig. Dis. Sci..

[126] Yi D., Liu W., Hou Y., Wang L., Zhao D., Wu T., Ding B., Guoyao W. (2018). Establishment of a Porcine Model of Indomethacin-Induced Intestinal Injury. Front. Biosci. Landmark.

[127] Khan A., Alsahli M., Rahmani A. (2018). Myeloperoxidase as an Active Disease Biomarker: Recent Biochemical and Pathological Perspectives. Med. Sci..

[128] Olza J., Aguilera C.M., Gil-Campos M., Leis R., Bueno G., Martínez-Jiménez M.D., Valle M., Canẽte R., Tojo R., Moreno L.A. (2012). Myeloperoxidase Is an Early Biomarker of Inflammation and Cardiovascular Risk in Prepubertal Obese Children. Diabetes Care.

[129] Schindhelm R.K., van der Zwan L.P., Teerlink T., Scheffer P.G. (2009). Myeloperoxidase: A Useful Biomarker for Cardiovascular Disease Risk Stratification?. Clin. Chem..

[130] Berdowska A., Zwirska-Korczala K. (2001). Neopterin Measurement in Clinical Diagnosis. J. Clin. Pharm. Ther..

[131] Fuchs D., Weiss G., Wachter H. (1993). Neopterin, Biochemistry and Clinical Use as a Marker for Cellular Immune Reactions. Int. Arch. Allergy Immunol..

[132] Hoffmann G., Wirleitner B., Fuchs D. (2003). Potential Role of Immune System Activation-Associated Production of Neopterin Derivatives in Humans. Inflamm. Res..

[133] Husain N., Tokoro K., Popov J.M., Naides S.J., Kwasny M.J., Buchman A.L. (2013). Neopterin Concentration as an Index of Disease Activity in Crohn’s Disease and Ulcerative Colitis. J. Clin. Gastroenterol..

[134] Nancey S., Boschetti G., Moussata D., Cotte E., Peyras J., Cuerq C., Haybrard J., Charlois A.L., Mialon A., Chauvenet M. (2013). Neopterin Is a Novel Reliable Fecal Marker as Accurate as Calprotectin for Predicting Endoscopic Disease Activity in Patients with Inflammatory Bowel Diseases. Inflamm. Bowel Dis..

[135] Maršálek P., Svoboda M., Smutná M., Blahová J., Večerek V. (2011). Neopterin and Biopterin as Biomarkers of Immune System Activation Associated with Castration in Piglets. J. Anim. Sci..

[136] Maršálek P., Svoboda M., Bernardy J., Večerek V. (2015). Concentrations of Neopterin, Biopterin, and Cortisol Associated with Surgical Castration of Piglets with Lidocaine. Czech J. Anim. Sci..

[137] Murray M., Coughlan M.T., Gibbon A., Kumar V., Marques F.Z., Selby-Pham S., Snelson M., Tsyganov K., Williamson G., Woodruff T.M. (2022). Reduced Growth, Altered Gut Microbiome and Metabolite Profile, and Increased Chronic Kidney Disease Risk in Young Pigs Consuming a Diet Containing Highly Resistant Protein. Front. Nutr..

[138] Altindag Z.Z., Baydar T., Isimer A., Sahin G. (2003). Neopterin as a New Biomarker for the Evaluation of Occupational Exposure to Silica. Int. Arch. Occup. Environ. Health.

[139] Firoz C.K., Jabir N.R., Kamal M.A., Alama M.N., Damanhouri G.A., Khan W., Alzahrani A.S., Almehdar H.A., Tabrez S. (2015). Neopterin: An Immune Biomarker of Coronary Artery Disease and Its Association with Other CAD Markers. IUBMB Life.

[140] Baydar T., Yuksel O., Sahin T.T., Dikmen K., Girgin G., Sipahi H., Kurukahvecioglu O., Bostanci H., Sare M. (2009). Neopterin as a Prognostic Biomarker in Intensive Care Unit Patients. J. Crit. Care.

[141] Melichar B., Spisarová M., Bartoušková M., Krcmová L.K., Javorská L., Študentová H. (2017). Neopterin as a Biomarker of Immune Response in Cancer Patients. Ann. Transl. Med..

[142] de Rosa S., Cirillo P., Pacileo M., Petrillo G., D’ascoli G.-L., Maresca F., Ziviello F., Chiariello M. (2011). Neopterin: From Forgotten Biomarker to Leading Actor in Cardiovascular Pathophysiology. Curr. Vasc. Pharmacol..

[143] Glatz J.F.C., van der Vusse G.J. (1996). Cellular fatty acid-binding proteins: Their function and physiological significance. Prog. Lipid Res..

[144] Pott J., Hornef M. (2012). Innate Immune Signalling at the Intestinal Epithelium in Homeostasis and Disease. EMBO Rep..

[145] Lau E., Marques C., Pestana D., Santoalha M., Carvalho D., Freitas P., Calhau C. (2016). The Role of I-FABP as a Biomarker of Intestinal Barrier Dysfunction Driven by Gut Microbiota Changes in Obesity. Nutr. Metab..

[146] Pelsers M.M.A.L., Namiot Z., Kisielewski W., Namiot A., Januszkiewicz M., Hermens W.T., Glatz J.F.C. (2003). Intestinal-Type and Liver-Type Fatty Acid-Binding Protein in the Intestine. Tissue Distribution and Clinical Utility. Clin. Biochem..

[147] Ockner R.K., Manning J.A. (1974). Fatty Acid Binding Protein in Small Intestine. Identification, Isolation, and Evidence for Its Role in Cellular Fatty Acid Transport. J. Clin. Investig..

[148] Kano H., Okada K., Morimoto K., Bao W., Fukase K., Ito A., Okita Y. (2015). Prediction of Reversibility of Intestinal Mucosal Damage after Ischemia-Reperfusion Injury by Plasma Intestinal Fatty Acid-Binding Protein Levels in Pigs. Perfusion.

[149] López-Colom P., Yu K., Barba-Vidal E., Saco Y., Martín-Orúe S.M., Castillejos L., Solà-Oriol D., Bassols A. (2019). I-FABP, Pig-MAP and TNF-α as Biomarkers for Monitoring Gut-Wall Integrity in Front of Salmonella Typhimurium and ETEC K88 Infection in a Weaned Piglet Model. Res. Vet. Sci..

[150] Niewold T.A., Meinen M., van der Meulen J. (2004). Plasma Intestinal Fatty Acid Binding Protein (I-FABP) Concentrations Increase Following Intestinal Ischemia in Pigs. Res. Vet. Sci..

[151] Zhang Q., Wu T., Li S., Meng Y., Tan Z., Wu M., Yi D., Wang L., Zhao D., Hou Y. (2021). Protective Effect of Zinc Oxide and Its Association with Neutrophil Degranulation in Piglets Infected with Porcine Epidemic Diarrhea Virus. Oxid. Med. Cell Longev..

[152] Liu Y., Xu Q., Wang Y., Liang T., Li X., Wang D., Wang X., Zhu H., Xiao K. (2021). Necroptosis Is Active and Contributes to Intestinal Injury in a Piglet Model with Lipopolysaccharide Challenge. Cell Death Dis..

[153] Güzel M., Sözüer E.M., Salt Ö., İkizceli İ., Akdur O., Yazıcı C. (2014). The Value of the Serum I-FABP Level for Diagnosing Acute Mesenteric Ischemia. Surg. Today.

[154] Cahyaningsih U., Satyaningtijas A.S., Tarigan R., Nugraha A.B. (2018). Chicken I-FABP as Biomarker of Chicken Intestinal Lesion Caused by Coccidiosis. IOP Conf. Ser. Earth Environ. Sci..

[155] Li L., Wang M., Chen J., Xu Z., Wang S., Xia X., Liu D., Wang S., Xie C., Wu J. (2021). Preventive Effects of Bacillus Licheniformis on Heat Stroke in Rats by Sustaining Intestinal Barrier Function and Modulating Gut Microbiota. Front. Microbiol..

[156] McGrath A.P., Hilmer K.M., Collyer C.A., Shepard E.M., Elmore B.O., Brown D.E., Dooley D.M., Guss J.M. (2009). Structure and Inhibition of Human Diamine Oxidase. Biochemistry.

[157] Luk G.D., Bayless T.M., Baylin S.B. (1983). Plasma Postheparin Diamine Oxidase Sensitive Provocative Test for Quantitating Length of Acute Intestinal Mucosal Injury in the Rat. J. Clin. Investig..

[158] Wolvekamp M.C.J., Bruin R.W.F. (1994). Diamine Oxidase: An Overview of Historical, Biochemical and Functional Aspects. Dig. Dis..

[159] Dieryck I., de Backere J., Paeshuyse J. (2022). Effect of Hatching System and Prophylactic Antibiotic Use on Serum Levels of Intestinal Health Biomarker Diamine Oxidase in Broilers at an Early Age. Animal.

[160] Tsunooka N., Maeyama K., Nakagawa H., Doi T., Horiuchi A., Miyauchi K., Watanabe Y., Imagawa H., Kawachi K. (2006). Localization and Changes of Diamine Oxidase during Cardiopulmonary Bypass in Rabbits. J. Surg. Res..

[161] Song Z., Cheng K., Zhang L., Wang T. (2017). Dietary Supplementation of Enzymatically Treated Artemisia Annua Could Alleviate the Intestinal Inflammatory Response in Heat-Stressed Broilers. J. Therm. Biol..

[162] Zhang L., Zhang L., Zhan X., Zeng X., Zhou L., Cao G., Chen A., Yang C. (2016). Effects of Dietary Supplementation of Probiotic, Clostridium Butyricum, on Growth Performance, Immune Response, Intestinal Barrier Function, and Digestive Enzyme Activity in Broiler Chickens Challenged with *Escherichia coli* K88. J. Anim. Sci. Biotechnol..

[163] Liu N., Wang J.Q., Jia S.C., Chen Y.K., Wang J.P. (2018). Effect of Yeast Cell Wall on the Growth Performance and Gut Health of Broilers Challenged with Aflatoxin B 1 and Necrotic Enteritis. Poult. Sci..

[164] Liu Y., Chen F., Odle J., Lin X., Jacobi S.K., Zhu H., Wu Z., Hou Y. (2012). Fish Oil Enhances Intestinal Integrity and Inhibits TLR4 and NOD2 Signaling Pathways in Weaned Pigs after LPS Challenge. J. Nutr..

[165] Hou Y., Wang L., Zhang W., Yang Z., Ding B., Zhu H., Liu Y., Qiu Y., Yin Y., Wu G. (2012). Protective Effects of N-Acetylcysteine on Intestinal Functions of Piglets Challenged with Lipopolysaccharide. Amino Acids.

[166] Wang L., Zhou J., Hou Y., Yi D., Ding B., Xie J., Zhang Y., Chen H., Wu T., Zhao D. (2017). N-Acetylcysteine Supplementation Alleviates Intestinal Injury in Piglets Infected by Porcine Epidemic Diarrhea Virus. Amino Acids.

[167] Zhang J., Zhao D., Yi D., Wu M., Chen H., Wu T., Zhou J., Li P., Hou Y., Wu G. (2019). Microarray Analysis Reveals the Inhibition of Intestinal Expression of Nutrient Transporters in Piglets Infected with Porcine Epidemic Diarrhea Virus. Sci. Rep..

[168] Çakmaz R., Büyükaşik O., Kahramansoy N., Erkol H., Çöl C., Boran Ç., Buǧdayci G. (2013). A Combination of Plasma DAO and Citrulline Levels as a Potential Marker for Acute Mesenteric Ischemia. Libyan J. Med..

[169] Wang S., Yang J., Zhang B., Wu K., Yang A., Li C., Zhang J., Zhang C., Rajput S.A., Zhang N. (2018). Deoxynivalenol Impairs Porcine Intestinal Host Defense Peptide Expression in Weaned Piglets and IPEC-J2 Cells. Toxins.

[170] Crenn P., Messing B., Cynober L. (2008). Citrulline as a Biomarker of Intestinal Failure Due to Enterocyte Mass Reduction. Clin. Nutr..

[171] Wu G., Knabe D.A., Flynn N.E. (1994). Synthesis of Citrulline from Glutamine in Pig Enterocytes. Biochem. J..

[172] Barzał J.A., Szczylik C., Rzepecki P., Jaworska M., Anuszewska E. (2014). Plasma Citrulline Level as a Biomarker for Cancer Therapy-Induced Small Bowel Mucosal Damage. Acta Biochim. Pol..

[173] Ye F., Ning J., Fardous Z., Katsube T., Li Q., Wang B. (2020). Citrulline, A Potential Biomarker of Radiation-Induced Small Intestine Damage. Dose-Response.

[174] van der Velden W.J.F.M., Herbers A.H.E., Brüggemann R.J.M., Feuth T., Peter Donnelly J., Blijlevens N.M.A. (2013). Citrulline and Albumin as Biomarkers for Gastrointestinal Mucositis in Recipients of Hematopoietic SCT. Bone Marrow Transpl..

[175] Lutgens L., Lambin P., Com W. (2007). Biomarkers for Radiation-Induced Small Bowel Epithelial Damage: An Emerging Role for Plasma Citrulline. World J. Gastroenterol..

[176] Jäckel S., Pipp F.C., Emde B., Weigt S., Vigna E., Hanschke B., Kasper L., Siddharta A., Hellmann J., Czasch S. (2021). L-Citrulline: A Preclinical Safety Biomarker for the Small Intestine in Rats and Dogs in Repeat Dose Toxicity Studies. J. Pharmacol. Toxicol. Methods.

[177] Shin S., Jeong H.M., Chung S.E., Kim T.H., Thapa S.K., Lee D.Y., Song C.H., Lim J.Y., Cho S.M., Nam K.Y. (2019). Simultaneous Analysis of Acetylcarnitine, Proline, Hydroxyproline, Citrulline, and Arginine as Potential Plasma Biomarkers to Evaluate NSAIDs-Induced Gastric Injury by Liquid Chromatography–Tandem Mass Spectrometry. J. Pharm. Biomed. Anal..

[178] Sacoor C., Barros L.M., Montezinho L. (2020). What Are the Potential Biomarkers That Should Be Considered in Diagnosing and Managing Canine Chronic Inflammatory Enteropathies?. Open Vet. J..

[179] Gerou-Ferriani M., Allen R., Noble P.J.M., German A.J., Caldin M., Batchelor D.J. (2018). Determining Optimal Therapy of Dogs with Chronic Enteropathy by Measurement of Serum Citrulline. J. Vet. Intern. Med..

[180] Fasano A. (2012). Zonulin, Regulation of Tight Junctions, and Autoimmune Diseases. Ann. N. Y. Acad. Sci..

[181] Vanuytsel T., Vermeire S., Cleynen I. (2013). The Role of Haptoglobin and Its Related Protein, Zonulin, in Inflammatory Bowel Disease. Tissue Barriers.

[182] el Asmar R., Panigrahi P., Bamford P., Berti I., Not T., Coppa G.V., Catassi C., Fasano A. (2002). Host-Dependent Zonulin Secretion Causes the Impairment of the Small Intestine Barrier Function after Bacterial Exposure. Gastroenterology.

[183] Clemente M.G., de Virgiliis S., Kang J.S., Macatagney R., Musu M.P., di Pierro M.R. (2003). Early Effects of Gliadin on Enterocyte Intracellular Signalling Involved in Intestinal Barrier Function. Gut.

[184] Fasano A. (2012). Intestinal Permeability and Its Regulation by Zonulin: Diagnostic and Therapeutic Implications. Clin. Gastroenterol. Hepatol..

[185] Malíčková K., Francová I., Lukáš M., Kolář M., Králíková E., Bortlík M., Ďuricová D., Štěpánková L., Zvolská K., Pánková A. (2017). Fecal Zonulin Is Elevated in Crohn’s Disease and in Cigarette Smokers. Pract. Lab. Med..

[186] Szymanska E., Wierzbicka A., Dadalski M., Kierkus J. (2021). Fecal Zonulin as a Noninvasive Biomarker of Intestinal Permeability in Pediatric Patients with Inflammatory Bowel Diseases—Correlation with Disease Activity and Fecal Calprotectin. J. Clin. Med..

[187] Rossi G., Gavazza A., Vincenzetti S., Mangiaterra S., Galosi L., Marchegiani A., Pengo G., Sagratini G., Ricciutelli M., Cerquetella M. (2021). Clinicopathological and Fecal Proteome Evaluations in 16 Dogs Presenting Chronic Diarrhea Associated with Lymphangiectasia. Vet. Sci..

[188] Meineri G., Martello E., Atuahene D., Miretti S., Stefanon B., Sandri M., Biasato I., Corvaglia M.R., Ferrocino I., Cocolin L.S. (2022). Effects of Saccharomyces Boulardii Supplementation on Nutritional Status, Fecal Parameters, Microbiota, and Mycobiota in Breeding Adult Dogs. Vet. Sci..

[189] Ohlsson B., Roth B., Larsson E., Höglund P. (2017). Calprotectin in Serum and Zonulin in Serum and Feces Are Elevated after Introduction of a Diet with Lower Carbohydrate Content and Higher Fiber, Fat and Protein Contents. Biomed. Rep..

[190] Damms-Machado A., Louis S., Schnitzer A., Volynets V., Rings A., Basrai M., Bischoff S.C. (2017). Gut Permeability Is Related to Body Weight, Fatty Liver Disease, and Insulin Resistance in Obese Individuals Undergoing Weight Reduction. Am. J. Clin. Nutr..

[191] Xiong W., Ma H., Zhang Z., Jin M., Wang J., Xu Y., Wang Z. (2019). Icariin Enhances Intestinal Barrier Function by Inhibiting NF-ΚB Signaling Pathways and Modulating Gut Microbiota in a Piglet Model. RSC Adv..

[192] Xu Y., Li Y., Scott K., Lindh C.H., Jakobsson K., Fletcher T., Ohlsson B., Andersson E.M. (2020). Inflammatory Bowel Disease and Biomarkers of Gut Inflammation and Permeability in a Community with High Exposure to Perfluoroalkyl Substances through Drinking Water. Environ. Res..

[193] Raetz C.R.H., Whitfield C. (2002). Lipopolysaccharide Endotoxins. Annu. Rev. Biochem..

[194] Sulc R., Szekely G., Shinde S., Wierzbicka C., Vilela F., Bauer D., Sellergren B. (2017). Phospholipid Imprinted Polymers as Selective Endotoxin Scavengers. Sci. Rep..

[195] Cheng C.S., Wei H.K., Wang P., Yu H.C., Zhang X.M., Jiang S.W., Peng J. (2019). Early Intervention with Faecal Microbiota Transplantation: An Effective Means to Improve Growth Performance and the Intestinal Development of Suckling Piglets. Animal.

[196] Xiong W., Huang J., Li X., Zhang Z., Jin M., Wang J., Xu Y., Wang Z. (2020). Icariin and Its Phosphorylated Derivatives Alleviate Intestinal Epithelial Barrier Disruption Caused by Enterotoxigenic *Escherichia coli* through Modulate P38 MAPK in Vivo and in Vitro. FASEB J..

[197] Hall D.M., Buettner G.R., Oberley L.W., Xu L., Matthes R.D., Gisolfi C.V., Hall D.M. (2001). Mechanisms of Circulatory and Intestinal Barrier Dysfunction during Whole Body Hyperthermia. Am. J. Physiol. Heart Circ. Physiol..

[198] Liu B., Zhu X., Cui Y., Wang W., Liu H., Li Z., Guo Z., Ma S., Li D., Wang C. (2022). Consumption of Dietary Fiber from Different Sources during Pregnancy Alters Sow Gut Microbiota and Improves Performance and Reduces Inflammation in Sows and Piglets. mSystems.

[199] Mokkala K., Pellonperä O., Röytiö H., Pussinen P., Rönnemaa T., Laitinen K. (2017). Increased Intestinal Permeability, Measured by Serum Zonulin, Is Associated with Metabolic Risk Markers in Overweight Pregnant Women. Metabolism.

[200] Ewaschuk J.B., Naylor J.M., Zello G.A. (2005). D-Lactate in Human and Ruminant Metabolism. J. Nutr..

[201] Montagnana M., Danese E., Lippi G. (2018). Biochemical Markers of Acute Intestinal Ischemia: Possibilities and Limitations. Ann. Transl. Med..

[202] Shi H., Wu B., Wan J., Liu W., Su B. (2015). The Role of Serum Intestinal Fatty Acid Binding Protein Levels and D-Lactate Levels in the Diagnosis of Acute Intestinal Ischemia. Clin. Res. Hepatol. Gastroenterol..

[203] Nielsen C., Kirkegård J., Erlandsen E.J., Lindholt J.S., Mortensen F.v. (2015). D-Lactate Is a Valid Biomarker of Intestinal Ischemia Induced by Abdominal Compartment Syndrome. J. Surg. Res..

[204] Nielsen C., Lindholt J.S., Erlandsen E.J., Mortensen F.v. (2011). D-Lactate as a Marker of Venous-Induced Intestinal Ischemia: An Experimental Study in Pigs. Int. J. Surg..

[205] Zhao W., Yuan M., Li P., Yan H., Zhang H., Liu J. (2019). Short-Chain Fructo-Oligosaccharides Enhances Intestinal Barrier Function by Attenuating Mucosa Inflammation and Altering Colonic Microbiota Composition of Weaning Piglets. Ital. J. Anim. Sci..

[206] Wang Y., Wang W., Wang R., Hao X., Duan Y., Meng Z., An X., Qi J. (2020). Dietary Fermented Soybean Meal Inclusion Improves Growth Performance and Ileal Barrier Function of the Weaned Piglets Challenged by Enterotoxigenic *Escherichia coli* K88. Anim. Feed Sci. Technol..

[207] Wu M., Xiao H., Ren W., Yin J., Tan B., Liu G., Li L., Nyachoti C.M., Xiong X., Wu G. (2014). Therapeutic Effects of Glutamic Acid in Piglets Challenged with Deoxynivalenol. PLoS ONE.

[208] Zhang H., Chen Y., Chen Y., Ji S., Jia P., Li Y., Wang T. (2020). Comparison of the Protective Effects of Resveratrol and Pterostilbene against Intestinal Damage and Redox Imbalance in Weanling Piglets. J. Anim. Sci. Biotechnol..

[209] Prakash N., Stumbles P., Mansfield C.S. (2019). Concentrations of Interleukin-6, -8, -10 and Tumour Necrosis Factor-α in the Faeces of Dogs with Acute Diarrhoea. N. Z. Vet. J..

[210] El M., Zaki S., Latif Alsayed M.A., Shrief R. (2020). Study of the Diagnostic Value of Interleukin-6 and Interleukin-8 in Children with Acute Gastroenteritis. Germs.

[211] El Feghaly R.E., Stauber J.L., Tarr P.I., Haslam D.B. (2013). Intestinal Inflammatory Biomarkers and Outcome in Pediatric Clostridium Difficile Infections. J. Pediatr..

[212] O’Neill L.A.J., Kishton R.J., Rathmell J. (2016). A Guide to Immunometabolism for Immunologists. Nat. Rev. Immunol..

[213] Donohoe D.R., Bultman S.J. (2012). Metaboloepigenetics: Interrelationships between Energy Metabolism and Epigenetic Control of Gene Expression. J. Cell Physiol..

[214] Shen W., Gao C., Cueto R., Liu L., Fu H., Shao Y., Yang W.Y., Fang P., Choi E.T., Wu Q. (2020). Homocysteine-Methionine Cycle Is a Metabolic Sensor System Controlling Methylation-Regulated Pathological Signaling. Redox Biol..

[215] Jung J., Zeng H., Horng T. (2019). Metabolism as a Guiding Force for Immunity. Nat. Cell Biol..

[216] Soto-Heredero G., Gómez de las Heras M.M., Gabandé-Rodríguez E., Oller J., Mittelbrunn M. (2020). Glycolysis–a Key Player in the Inflammatory Response. FEBS J..

[217] Zasłona Z., O’Neill L.A.J. (2020). Cytokine-like Roles for Metabolites in Immunity. Mol. Cell.

[218] Chauhan P., Saha B. (2018). Metabolic Regulation of Infection and Inflammation. Cytokine.

[219] McGettrick A.F., O’Neill L.A.J. (2020). The Role of HIF in Immunity and Inflammation. Cell Metab..

[220] Pålsson-McDermott E.M., O’Neill L.A.J. (2020). Targeting Immunometabolism as an Anti-Inflammatory Strategy. Cell Res..

[221] Ryan D.G., O’neill L.A.J. (2020). Krebs Cycle Reborn in Macrophage Immunometabolism. Annu. Rev. Immunol..

[222] Harber K.J., de Goede K.E., Verberk S.G.S., Meinster E., de Vries H.E., van Weeghel M., de Winther M.P.J., van den Bossche J. (2020). Succinate Is an Inflammation-Induced Immunoregulatory Metabolite in Macrophages. Metabolites.

[223] Fortuny L., Sebastián C. (2021). Sirtuins as Metabolic Regulators of Immune Cells Phenotype and Function. Genes.

[224] Peace C.G., O’Neill L.A.J. (2022). The Role of Itaconate in Host Defense and Inflammation. J. Clin. Investig..

[225] Masson N., Ratcliffe P.J. (2014). Hypoxia Signaling Pathways in Cancer Metabolism: The Importance of Co-Selecting Interconnected Physiological Pathways. Cancer Metab..

[226] Corcoran S.E., O’Neill L.A.J. (2016). HIF1α and Metabolic Reprogramming in Inflammation. J. Clin. Investig..

[227] Katayama K., Wada K., Nakajima A., Mizuguchi H., Hayakawa T., Nakagawa S., Kadowaki T., Nagai R., Kamisaki Y., Blumberg R.S. (2003). Basic-Alimentary Tract A Novel PPAR Gene Therapy to Control Inflammation Associated with Inflammatory Bowel Disease in a Murine Model. Gastroenterology.

[228] Croasdell A., Duffney P.F., Kim N., Lacy S.H., Sime P.J., Phipps R.P. (2015). PPAR γ and the Innate Immune System Mediate the Resolution of Inflammation. PPAR Res..

[229] Mauro C., Leow S.C., Anso E., Rocha S., Thotakura A.K., Tornatore L., Moretti M., de Smaele E., Beg A.A., Tergaonkar V. (2011). NF-ΚB Controls Energy Homeostasis and Metabolic Adaptation by Upregulating Mitochondrial Respiration. Nat. Cell Biol..

[230] Johnson R.F., Perkins N.D. (2012). Nuclear Factor-ΚB, P53, and Mitochondria: Regulation of Cellular Metabolism and the Warburg Effect. Trends Biochem. Sci..

[231] Mills E., O’Neill L.A.J. (2014). Succinate: A Metabolic Signal in Inflammation. Trends Cell Biol..

[232] Macias-Ceja D.C., Ortiz-Masiá D., Salvador P., Gisbert-Ferrándiz L., Hernández C., Hausmann M., Rogler G., Esplugues J.V., Hinojosa J., Alós R. (2019). Succinate Receptor Mediates Intestinal Inflammation and Fibrosis. Mucosal. Immunol..

[233] Zotta A., Zaslona Z., O’Neill L.A. (2020). Is Citrate A Critical Signal in Immunity and Inflammation?. J. Cell. Signal. Rev. Artic..

[234] Navarro M.N., Gómez de las Heras M.M., Mittelbrunn M. (2022). Nicotinamide Adenine Dinucleotide Metabolism in the Immune Response, Autoimmunity and Inflammageing. Br. J. Pharmacol..

[235] Rongvaux A., Shea R.J., Mulks M.H., Gigot D., Urbain J., Leo O., Andris F. (2002). Pre-B-Cell Colony-Enhancing Factor, Whose Expression Is up-Regulated in Activated Lymphocytes, Is a Nicotinamide Phosphoribosyltransferase, a Cytosolic Enzyme Involved in NAD Biosynthesis. Eur. J. Immunol..

[236] Gerner R.R., Klepsch V., Macheiner S., Arnhard K., Adolph T.E., Grander C., Wieser V., Pfister A., Moser P., Hermann-Kleiter N. (2018). NAD Metabolism Fuels Human and Mouse Intestinal Inflammation. Gut.

[237] Vachharajani V.T., Liu T., Wang X., Hoth J.J., Yoza B.K., McCall C.E. (2016). Sirtuins Link Inflammation and Metabolism. J. Immunol. Res..

[238] Kogut M.H., Genovese K.J., He H., Arsenault R.J. (2016). AMPK and MTOR: Sensors and Regulators of Immunometabolic Changes during Salmonella Infection in the Chicken. Poult. Sci..

[239] Proszkowiec-Weglarz M., Richards M.P. (2007). 5′-AMP-Activated Protein Kinase in Avian Biology. Avian Poult. Biol. Rev..

[240] O’Neill L.A.J., Grahame Hardie D. (2013). Metabolism of Inflammation Limited by AMPK and Pseudo-Starvation. Nature.

[241] Yang C.S., Kim J.J., Lee H.M., Jin H.S., Lee S.H., Park J.H., Kim S.J., Kim J.M., Han Y.M., Lee M.S. (2014). The AMPK-PPARGC1A Pathway Is Required for Antimicrobial Host Defense through Activation of Autophagy. Autophagy.

[242] Blagih J., Coulombe F., Vincent E.E., Dupuy F., Galicia-Vázquez G., Yurchenko E., Raissi T.C., van der Windt G.J.W., Viollet B., Pearce E.L. (2015). The Energy Sensor AMPK Regulates T Cell Metabolic Adaptation and Effector Responses In Vivo. Immunity.

[243] Laplante M., Sabatini D.M. (2012). MTOR Signaling in Growth Control and Disease. Cell.

[244] Cobbold S.P. (2013). The MTOR Pathway and Integrating Immune Regulation. Immunology.

[245] Powell J.D., Pollizzi K.N., Heikamp E.B., Horton M.R. (2012). Regulation of Immune Responses by MTOR. Annu. Rev. Immunol..

[246] Battaglioni S., Benjamin D., Wälchli M., Maier T., Hall M.N. (2022). MTOR Substrate Phosphorylation in Growth Control. Cell.

